# A split influenza vaccine formulated with a combination adjuvant composed of alpha-d-glucan nanoparticles and a STING agonist elicits cross-protective immunity in pigs

**DOI:** 10.1186/s12951-022-01677-2

**Published:** 2022-11-11

**Authors:** V. Patil, J. F. Hernandez-Franco, G. Yadagiri, D. Bugybayeva, S. Dolatyabi, N. Feliciano-Ruiz, J. Schrock, J. Hanson, J. Ngunjiri, H. HogenEsch, G. J. Renukaradhya

**Affiliations:** 1 Center for Food Animal Health, Department of Animal Sciences, The Ohio State University, 1680 Madison Avenue, Wooster, OH 44691 USA; 2 Department of Comparative Pathobiology, College of Veterinary Medicine, Purdue University, West Lafayette, IN USA; 3 International Center for Vaccinology, Kazakh National Agrarian Research University (KazNARU), Almaty, Kazakhstan

**Keywords:** Nano-11, ADU-S100, Swine influenza A viruses, Vaccination, Cellular immunity, Pigs

## Abstract

**Background:**

Swine influenza A viruses (SwIAVs) pose an economic and pandemic threat, and development of novel effective vaccines is of critical significance. We evaluated the performance of split swine influenza A virus (SwIAV) H1N2 antigens with a plant-derived nanoparticle adjuvant alone (Nano-11) [Nano11-SwIAV] or in combination with the synthetic stimulator of interferon genes (STING) agonist ADU-S100 (NanoS100-SwIAV). Specific pathogen free (SPF) pigs were vaccinated twice via intramuscular (IM) or intradermal (ID) routes and challenged with a virulent heterologous SwIAV H1N1-OH7 virus.

**Results:**

Animals vaccinated IM or ID with NanoS100-SwIAV had significantly increased cross-reactive IgG and IgA titers in serum, nasal secretion and bronchoalveolar lavage fluid at day post challenge 6 (DPC6). Furthermore, NanoS100-SwIAV ID vaccinates, even at half the vaccine dose compared to their IM vaccinated counterparts, had significantly increased frequencies of CXCL10^+^ myeloid cells in the tracheobronchial lymph nodes (TBLN), and IFNγ^+^ effector memory T-helper/memory cells, IL-17A^+^ total T-helper/memory cells, central and effector memory T-helper/memory cells, IL-17A^+^ total cytotoxic T-lymphocytes (CTLs), and early effector CTLs in blood compared with the Nano11-SwIAV group demonstrating a potential dose-sparing effect and induction of a strong IL-17A^+^ T-helper/memory (Th17) response in the periphery. However, the frequencies of IFNγ^+^ late effector CTLs and effector memory T-helper/memory cells, IL-17A^+^ total CTLs, late effector CTLs, and CXCL10^+^ myeloid cells in blood, as well as lung CXCL10^+^ plasmacytoid dendritic cells were increased in NanoS100-SwIAV IM vaccinated pigs. Increased expression of IL-4 and IL-6 mRNA was observed in TBLN of Nano-11 based IM vaccinates following challenge. Furthermore, the challenge virus load in the lungs and nasal passage was undetectable in NanoS100-SwIAV IM vaccinates by DPC6 along with reduced macroscopic lung lesions and significantly higher virus neutralization titers in lungs at DPC6. However, NanoS100-SwIAV ID vaccinates exhibited significant reduction of challenge virus titers in nasal passages and a remarkable reduction of challenge virus in lungs.

**Conclusions:**

Despite vast genetic difference (77% HA gene identity) between the H1N2 and H1N1 SwIAV, the NanoS100 adjuvanted vaccine elicited cross protective cell mediated immune responses, suggesting the potential role of this combination adjuvant in inducing cross-protective immunity in pigs.

**Graphical Abstract:**

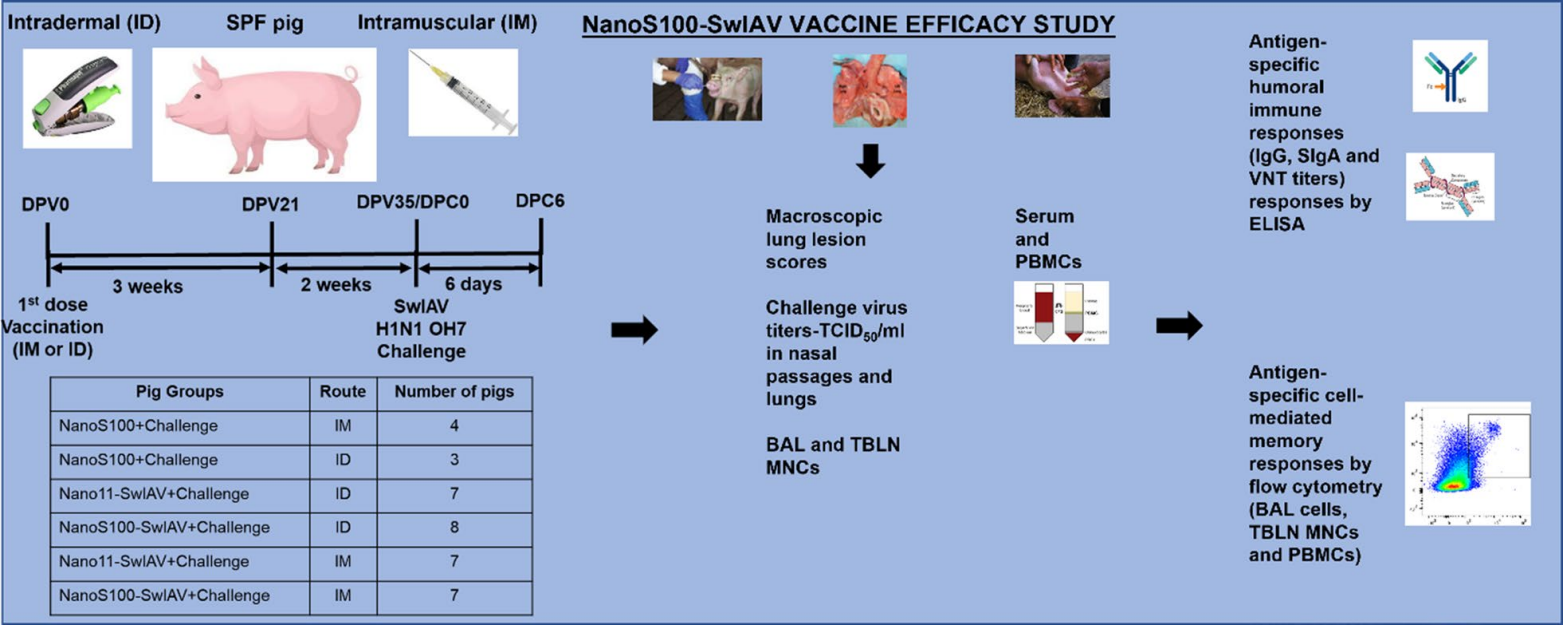

**Supplementary Information:**

The online version contains supplementary material available at 10.1186/s12951-022-01677-2.

## Introduction

Swine influenza A virus (SwIAV) is the causative agent of acute respiratory infection in pigs leading to substantial economic losses globally. There are three circulating subtypes of SwIAV, H1N1, H1N2, and H3N2, with significant heterogeneity in their genesis, genetic background, and antigenic characteristics. Pigs play a unique role either by being an intermediate host to both avian and mammalian influenza A viruses (IAVs) and as a ‘mixing vessel’ facilitating genetic reassortment and interspecies dissemination. Therefore, effective control and mitigation of SwIAV infection in swine herds is of great public health significance [1]. Hence, the development and characterization of novel effective vaccines conferring cross-protection and long-lasting immunity against evolving strains and subtypes is crucial.

Antigen-specific cell-mediated immune responses play a significant role in host defense against viral respiratory diseases. The crucial role played by antigen-specific T-cells in alleviating the severity and attenuation of virus shedding in humans has been demonstrated during the 2009 influenza pandemic [2, 3]. Even in the absence of neutralizing antibodies, influenza virus-specific T-cell responses are protective in mice [4]. Similarly in pigs, in the absence of neutralizing antibodies, vaccination with a signal minus Flu (S-FLU) universal influenza vaccine resulted in amelioration of lung pathology and decreased virus burden in nasal passage and lungs after the homologous and moderately matched virus challenge [5]. Furthermore, in pigs, intranasal delivery of SwIAV H1N1-OH7 viral peptides entrapped poly d,l-lactic-*co*-glycolic acid (PLGA) nanoparticles alleviated lung pathology following a heterologous challenge by stimulating strong antigen-specific T-cell responses but not neutralizing antibody responses [6]. The SwIAV H1N2-specific activated and cytokines secreting polyfunctional T-cell kinetics were characterized in pigs at various time points 4–44 days post infection in the lung draining tracheobronchial lymph nodes mononuclear cells (TBLN MNCs), in peripheral blood mononuclear cells (PBMCs) and bronchoalveolar lavage fluid (BAL) cells [7]. In that study, a SwIAV infection induced cell-mediated immune (CMI) responses were revealed in detail, and does not suggest a protective role of such a specific CMI response in vaccinated pigs. Therefore, in the present study, we determined the SwIAV-specific cell-mediated immune responses in detail in vaccinated pigs.

There is an urgent unmet demand for advanced vaccine technologies. Furthermore, diseases caused by existing and emerging viruses such as porcine epidemic diarrhea virus, influenza, and African swine fever, impose a significant economic burden on the global swine industry posing threat to food security and public health. Traditional approaches such as intramuscular administration of inactivated and modified live virus vaccines provide inadequate protection under field conditions and are associated with safety concerns. Alternative methods of vaccine delivery and new vaccine formulations are needed to augment protection against evolving virus strains and subtypes. Nanoparticle-based vaccine formulations offer significant advantages by virtue of their unique physicochemical properties. Their size facilitates uptake by dendritic cells (DCs) and allows administration via alternative routes such as intranasal, oral, or intradermal routes which can augment the immune response by targeting tissues rich in antigen-presenting cells and avoids the use of needles. Although the use of NPs in vaccines has tremendous potential, the adoption of synthetic NPs has been slowed by difficulties in scaling-up production, lack of stability and high cost. Plant-derived NPs may be able to overcome these challenges, for animal vaccines.

Alpha-d-glucans are polysaccharide polymers in plants, animals, and microbes. Plant-derived phytoglycogen (PG) is a densely branched dendrimer-like a-d-glucan that forms nanoparticle structures. Two simple chemical modifications of corn-derived PG create positively charged, amphiphilic nanoparticles, known as Nano-11, that stimulate immune responses when used as vaccine adjuvant in a variety of species [8, 9]. Nano-11 is a versatile adjuvant that can be used for alternative routes of vaccination and in combination with other immunostimulatory molecules. Interestingly, our methodology of two-step chemical modification confers intrinsic immunostimulatory activity to Nano-11 which appears to be unique to this preparation. Cationic alpha-d-glucan nanoparticles generated via different chemical modifications had no immunostimulatory activity by themselves [10, 11].

Nanoparticles (NPs)-based vaccines are effective in inducing cross-protective immune responses compared to their conventional inactivated whole virus multivalent vaccine counterparts [12, 13]. Such vaccines offer safe and efficacious alternative options against infectious diseases [14, 15]. Earlier research from our group characterized corn-based cationic alpha-d-glucan nanoparticles (Nano-11) and validated the performance as a dependable delivery platform in mice and pigs [9, 16–18]. Nano-11 has intrinsic adjuvanticity [9, 16], and the overall adjuvant effect can be further enhanced by incorporating additional immunostimulatory molecules. For example, the antigenicity of SwIAV killed antigen was augmented using a Nano-11 with Toll-like receptor-3 (TLR3) agonist poly(I:C) combination adjuvant [12, 17]. Cyclic dinucleotides are ligands of the intracellular STING molecule. Binding of STING activates the interferon regulatory transcription factor 3 (IRF3) and nuclear factor-ĸB (NF-κB) signaling pathways resulting in activation and maturation of antigen-presenting cells [19]. We showed previously that cyclic-di-adenosine monophosphate (AMP) readily adsorbs to Nano-11 and that the combination adjuvant enhances the immune response following intradermal immunization of mice and pigs with ovalbumin as a model antigen [20]. Inactivated IAV split antigens vaccines can offer better cross-protection than inactivated whole virus vaccines [21, 22]. The present study was initiated to characterize a novel SwIAV vaccine composed of inactivated split virus SwIAV H1N2-OH10 antigens with STING agonist ADU-S100, a synthetic analogue of cyclic-di-AMP, co-adsorbed onto Nano-11 (NanoS100-SwIAV) for use in pigs. Furthermore, we compared the effect of intramuscular (IM) injection with needle-free intradermal (ID) vaccine delivery.

## Results

### Characterization of the vaccine formulations

The adsorption of ADU-S100 to Nano-11 was 80–90% and was stable over 3 days (Table 1). Adsorption did not change the size of Nano-11, but slightly decreased the zeta potential and increased the overall particle size (Table 1). The adsorption of split viral antigens to Nano-11 and NanoS100 ranged from 53 to 64% between two different batches.

**Table 1:**
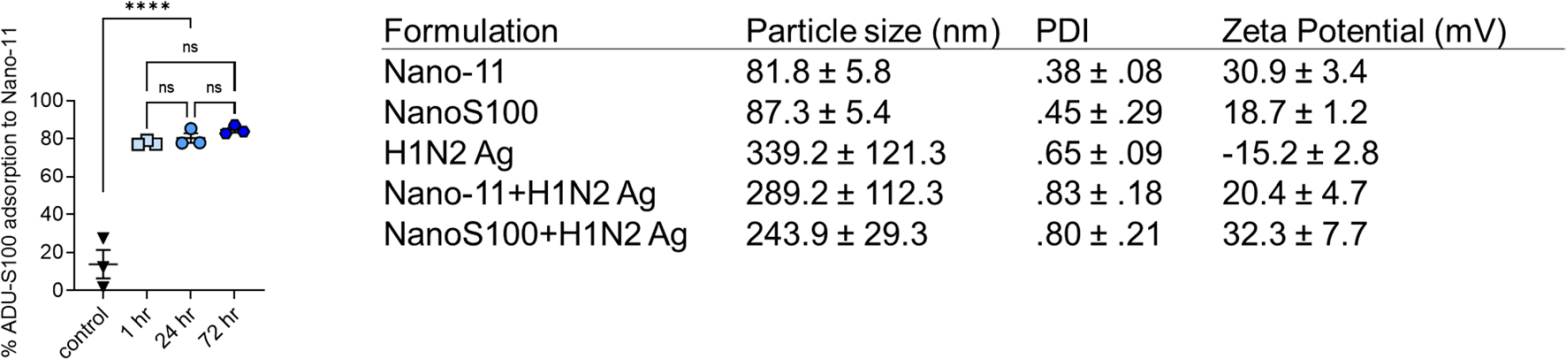
Characterization of Nano-11-based vaccine formulations used in the study

The table depicts the particle size (nm), PDI and Zeta potential (mV) of all the vaccine formulations used in the study

### The candidate SwIAV vaccines reduced the challenge virus load in the respiratory tract

**Fig. 1:**
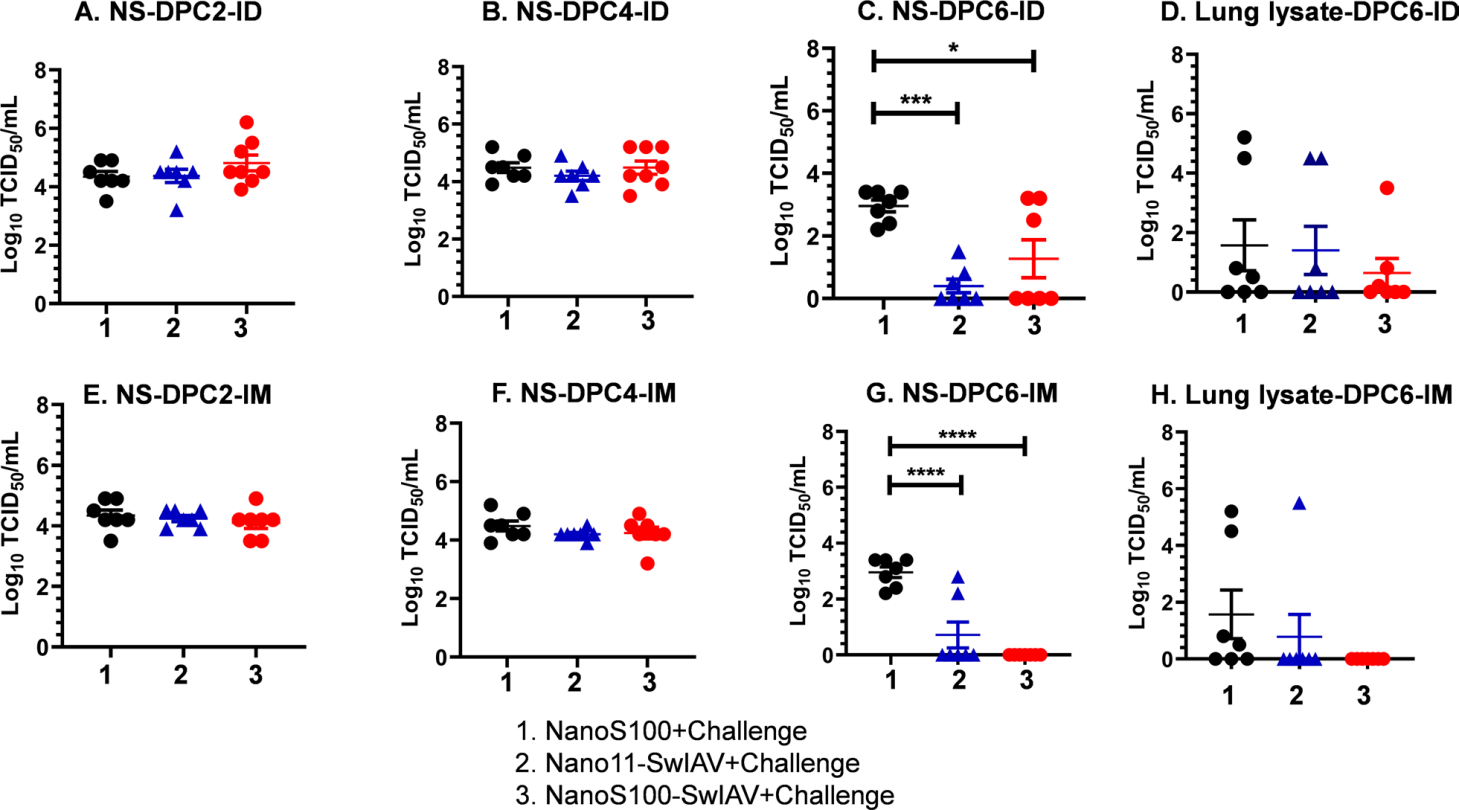
Infectious challenge virus load in nostrils and lungs of vaccinated pigs. Five-week-old SPF pigs were administered twice ID/IM with NanoS100-SwIAV, Nano11-SwIAV or control NanoS100 and challenged at post-prime vaccination day 35 with SwIAV H1N1-OH7. The challenge viral load in the respiratory tract was determined by cell culture technique in nasal swab (NS) at **A**, **E** DPC2, **B**, **F** DPC4, **C**, **G** DPC6, and **D**, **H** in lung lysate at DPC6 for ID and IM vaccinates, respectively. Data represent the mean value of 7–8 pigs ± SEM. Statistical analysis was performed by one-way ANOVA followed by Tukey’s post-test. Asterisk represents significant difference between indicated groups (*/♯ *p* < 0.05, **/♯♯ *p*< 0.01, and ***/♯♯♯ *p* < 0.001)

The protective efficacy of the Nano11-SwIAV and NanoS100-SwIAV vaccines administered IM or ID was assessed in pigs by measuring the titers of the SwIAV H1N1-OH7 challenge virus in the nasal swabs and lung lysates. At DPC2 (Fig. 1A and E) and DPC4 (Fig. 1B and F), there was no difference between the control group and vaccinated pigs. However, there was a significant reduction of virus titers in the nasal swabs of the vaccinated pigs on DPC6 (Fig. 1C and G). Nano11-SwIAV-ID vaccinates exhibited significant reduction in the challenge virus titers in nasal passages (*p* < 0.001) compared to NanoS100 mock group at DPC6 (Fig. 1C). Likewise, in case of NanoS100-SwIAV-ID vaccinates, a significant reduction of the challenge virus titers (*p* < 0.05) compared to the mock group animals was observed in the nasal passages at DPC6 (Fig. 1C). However, there was a significant reduction of challenge virus titers in nasal swabs in both Nano11-SwIAV-IM, NanoS100-SwIAV-IM vaccinates compared to their counterparts from the NanoS100 group (*p* < 0.01 to *p* < 0.001) at DPC6 (Fig. 1G). It should be noted that the ID vaccinated groups received half the dose of the IM vaccinated groups.

There was a trend towards a reduction of virus load in the lung lysates of vaccinated pigs on DPC6, but this did not reach statistical significance (Fig. 1D and H). In the lung lysates, the challenge virus titers in NanoS100-SwIAV-ID vaccinates, though statistically not significant, 6 out of 8 animals were able to effectively reduce the virus titers at DPC6 (Fig. 1D), while the virus was undetectable in NanoS100-SwIAV-IM vaccinates (Fig. 1H). Hence, the ID vaccinated groups significantly cleared the challenge SwIAV virus in nasal passages and substantially in lungs although they received half the dose of the IM vaccinated groups, indicating a potential dose-sparing effect. While Nano11-SwIAV vaccinates were able to significantly clear the challenge virus titers in nasal passages at DPC6, the addition of the STING agonist ADU-S100 resulted in more effective reduction of the challenge virus in NanoS100-SwIAV vaccinates. Overall, these findings confirm that the NanoS100-SwIAV vaccine offered protection in terms of reduced virus load at DPC6.

### Nano11-SwIAV and NanoS100-SwIAV increased the frequencies of innate CXCL10^+^ myeloid cells in mucosal and peripheral compartments of vaccinated pigs

**Fig. 2:**
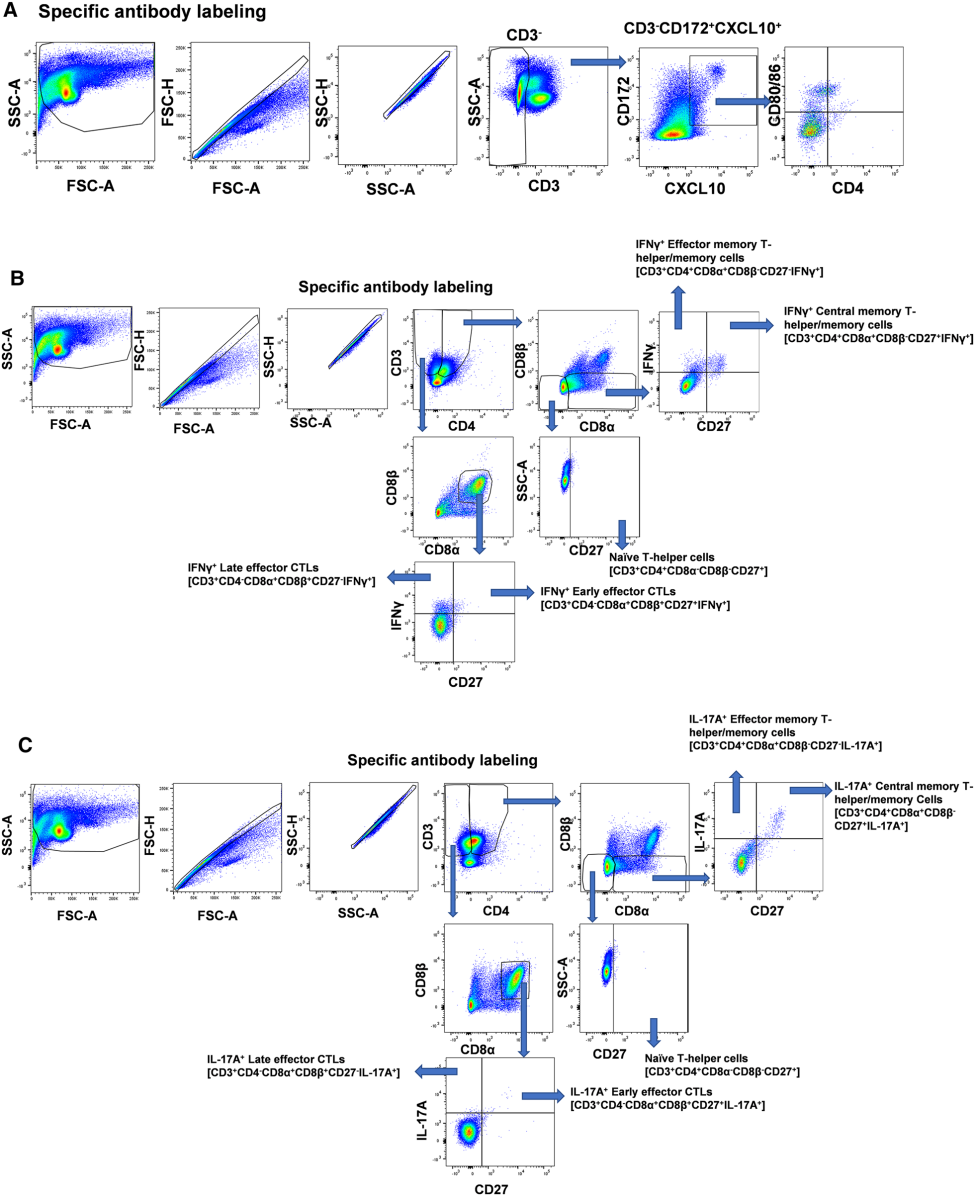
Representative gating strategy for the analysis of lymphocytes and myeloid cells in TBLN MNCs**.** Five-week-old SPF pigs were administered twice ID/IM with NanoS100-SwIAV, Nano11-SwIAV or control Nano11S100 and challenged at post-prime vaccination day 35 with SwIAV H1N1-OH7. PBMCs, TBLN MNCs, and BAL cells were isolated at DPC6 and were restimulated with SwIAV H1N1-OH7 in vitro. The cells were labeled and analyzed by flow cytometry for the frequencies of different types of myeloid and lymphocyte subsets. **A** CXCL10^+^ myeloid cells; **B** IFNγ^+^ early/late effector CTLs and central/effector memory T-helper/memory T-helper cells; **C** IL-17A^+^ early/late effector CTLs and central/effector memory T-helper/memory T-helper cells

To decipher the underlying mechanisms of protection, innate immune responses in TBLN MNCs, BAL cells, and PBMCs were investigated, and a representative gating pattern to delineate the different myeloid immune cells is shown in Fig. 2A. Chemokines mediate the recruitment of a variety of cells expressing cognate transmembrane G protein chemokine receptors. CXCL10 also referred to as IFNγ-inducible protein 10 is a chemokine expressed by different cells including myeloid cells. Cells of the adaptive immune system such as activated T-helper type 1 (Th1) lymphocytes express CXCR3, the receptor for CXCL10, and they are recruited to the sites of infection/inflammation at least in part by CXCL10-secreting myeloid cells [23]. Hence, we determined the frequencies of CXCL10^+^ myeloid cells in different immune compartments.

**Fig. 3:**
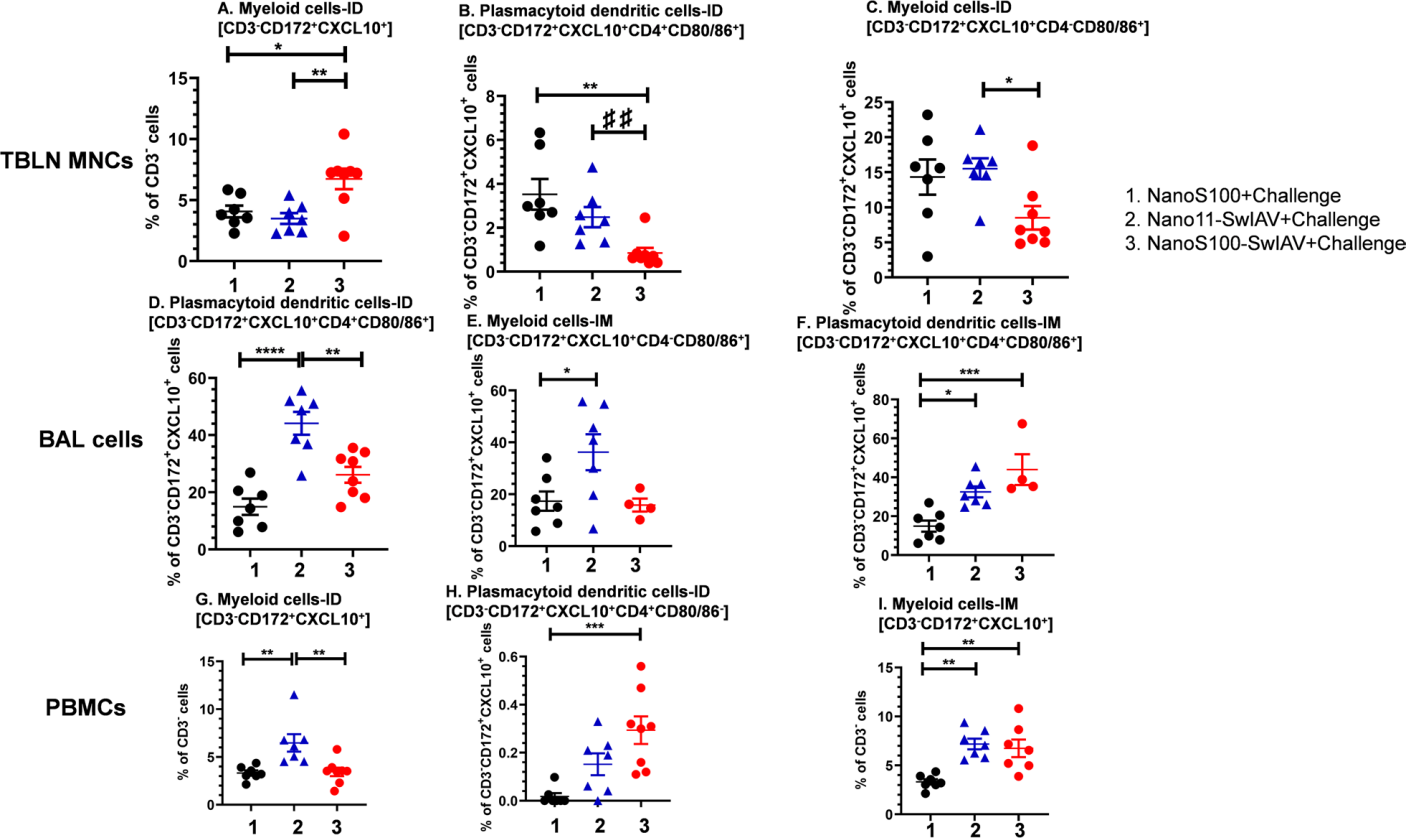
NanoS100-SwIAV modulated activated myeloid immune cell frequencies in vaccinated pigs. Five-week-old SPF pigs were administered twice ID/IM with NanoS100-SwIAV, Nano11-SwIAV or control NanoS100 and challenged at post-prime vaccination day 35 with SwIAV H1N1-OH7. TBLN MNCs, BAL cells, and PBMCs were isolated at DPC6 and were restimulated with SwIAV H1N1-OH7 in vitro. The cells were labeled and analyzed by flow cytometry for the frequencies of activated myeloid cell subsets: **A** Myeloid cells-ID [CD3^−^CD172^+^CXCL10^+^]; **B** Plasmacytoid dendritic cells-ID [CD3^−^CD172^+^CXCL10^+^CD4^+^CD80/86^+^]; **C** Myeloid cells-ID [CD3^−^CD172^+^CXCL10^+^CD4^−^CD80/86^+^] in TBLN MNCs; **D** Plasmacytoid dendritic cells-ID [CD3^−^CD172^+^CXCL10^+^CD4^+^CD80/86^+^]; **E** Myeloid cells-IM [CD3^−^CD172^+^CXCL10^+^CD4^−^CD80/86^+^]; **F** Plasmacytoid dendritic cells-IM [CD3^−^CD172^+^CXCL10^+^CD4^+^CD80/86^+^] in BAL cells; **G** Myeloid cells-ID [CD3^−^CD172^+^CXCL10^+^]; **H** Plasmacytoid dendritic cells-ID [CD3^−^CD172^+^CXCL10^+^CD4^+^CD80/86^−^]; and **I** Myeloid cells-IM [CD3^−^CD172^+^CXCL10^+^] in PBMCs. Data represent the mean value of 7–8 pigs ± SEM. Statistical analysis was performed by one-way ANOVA followed by Tukey’s post-test. For head-to-head comparison between two groups, Unpaired t-test was used. Asterisk/Pound represents significant difference between indicated groups (*/♯ *p* < 0.05, **/♯♯ *p*< 0.01, and ***/♯♯♯ *p* < 0.001)

The frequencies of CXCL10^+^ myeloid cells in TBLN MNCs were significantly increased in NanoS100-SwIAV-ID vaccinates compared to their counterparts from Nano11-SwIAV-ID and NanoS100 control groups (*p* < 0.05 to *p* < 0.01) (Fig. 3A). However, the frequencies of CD3^−^CD172^+^CXCL10^+^CD4^+^CD80/86^+^ plasmacytoid dendritic cells, and CD3^−^CD172^+^CXCL10^+^CD4^−^CD80/86^+^ myeloid cells were significantly reduced in NanoS100-SwIAV-ID vaccinates compared to Nano11-SwIAV-ID and the NanoS100 group (*p* < 0.01) (Fig. 3B and C) in TBLN. In case of BAL cells, NanoS100-SwIAV ID vaccinates exhibited a significant decrease of the frequencies of plasmacytoid dendritic cells compared to their counterparts from Nano11-SwIAV-ID group (*p* < 0.01) (Fig. 3D). However, Nano11-SwIAV vaccinates significantly upregulated the frequencies of plasmacytoid dendritic cells compared to the NanoS100 group (*p* < 0.0001) (Fig. 3D). Furthermore, the frequencies of activated plasmacytoid dendritic cells were significantly enhanced in both Nano11-SwIAV-IM and NanoS100-SwIAV-IM groups compared to NanoS100 vaccinates (*p* < 0.05 to *p* < 0.001) (Fig. 3F). The frequencies of activated myeloid cells was significantly increased in the Nano11-SwIAV-IM vaccinates compared to NanoS100 group (*p* < 0.05) (Fig. 3E) in BAL cells. Whereas in the peripheral compartment among PBMCs, the frequencies of CXCL10^+^ myeloid cells were significantly increased in Nano11-SwIAV-ID vaccinates compared to their counterparts from NanoS100-SwIAV-ID and NanoS100 groups (*p* < 0.01) (Fig. 3G). The frequencies of CXCL10^+^CD80/86^−^ plasmacytoid dendritic cells were significantly enhanced in the NanoS100-SwIAV-ID group animals compared to their NanoS100 counterparts (*p* < 0.001) (Fig. 3H). The vaccinates from both Nano11-SwIAV-IM, and NanoS100-SwIAV-IM groups significantly upregulated the frequencies of CXCL10^+^ myeloid cells compared to the NanoS100 group (*p* < 0.01) (Fig. 3I). Taken together, these results suggested that both Nano11-SwIAV and NanoS100-SwIAV vaccines elicited robust innate immune responses (CXCL10^+^ myeloid cells) in the lung draining TBLN, lungs and systemically in pigs at DPC6.

### Phenotypes of virus-specific adaptive immune cell types in the draining TBLN and lungs of vaccinated pigs at DPC6

**Fig. 4:**
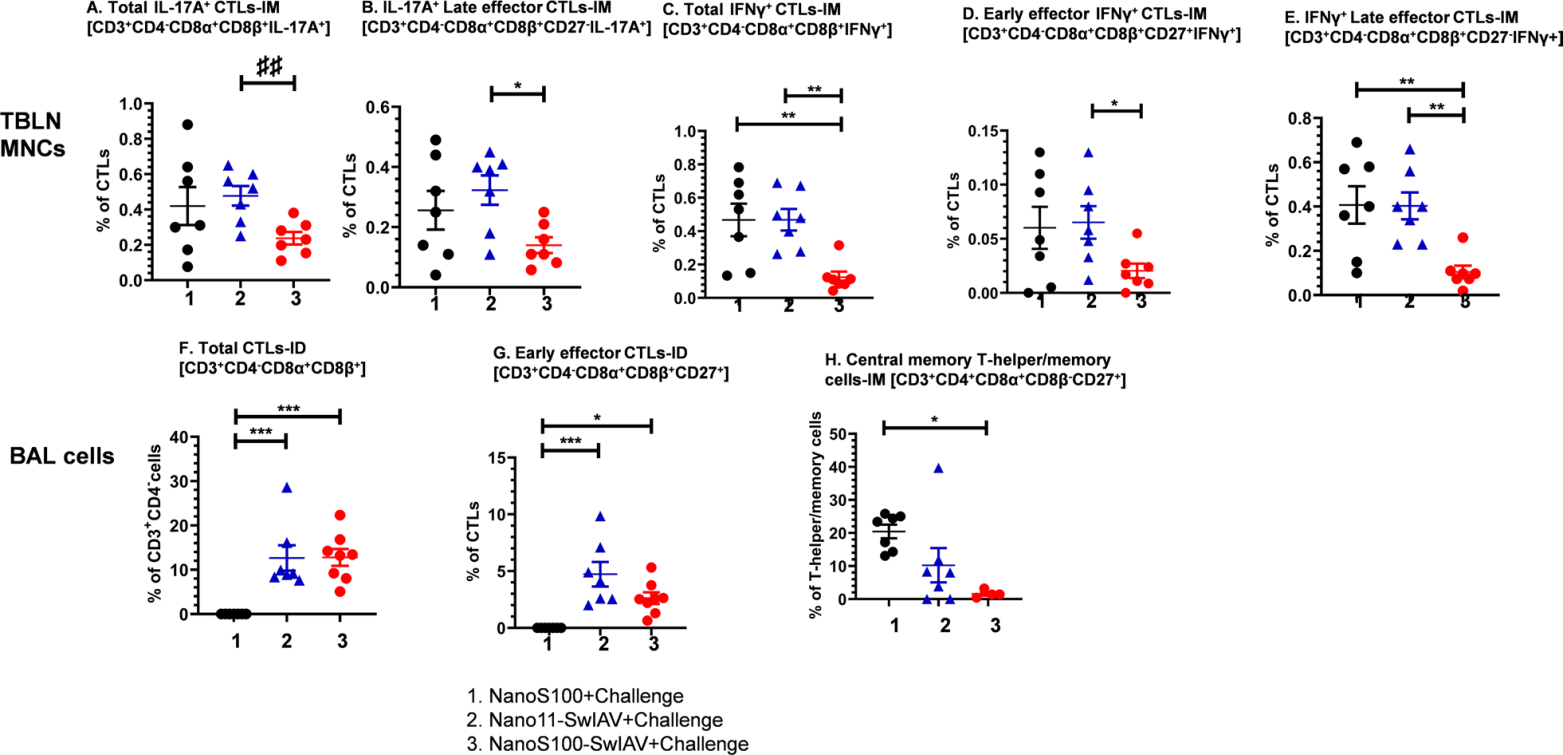
NanoS100-SwIAV altered the frequencies of T-lymphocytes in TBLN MNCs and BAL cells of vaccinated pigs. Five-week-old SPF pigs were administered twice ID/IM with NanoS100-SwIAV, Nano11-SwIAV or control NanoS100 and challenged at post-prime vaccination day 35 with SwIAV H1N1-OH7. TBLN MNCs and BAL cells were isolated at DPC6 and were restimulated with SwIAV H1N1-OH7 in vitro. The cells were labeled and analyzed by flow cytometry for the frequencies of T-lymphocyte subsets: **A** Total IL-17A^+^ CTLs-IM [CD3^+^CD4^−^CD8α^+^CD8β^+^IL-17A^+^]; **B** IL-17A^+^ Late effector CTLs-IM [CD3^+^CD4^−^CD8α^+^CD8β^+^CD27^−^IL-17A^+^]; **C** Total IFNγ^+^ CTLs-IM [CD3^+^CD4^−^CD8α^+^CD8β^+^IFNγ^+^]; **D** IFNγ^+^ Early effector CTLs-IM [CD3^+^CD4^−^CD8α^+^CD8β^+^CD27^+^IFNγ^+^]; **E** IFNγ^+^ Late effector CTLs-IM [CD3^+^CD4^−^CD8α^+^CD8β^+^CD27^−^IFNγ^+^] in TBLN MNCs; **F** Total CTLs-ID [CD3^+^CD4^−^CD8α^+^CD8β^+^]; **G** Early effector CTLs-ID [CD3^+^CD4^−^CD8α^+^CD8β^+^CD27^+^]; **H** Central memory T-helper/memory cells-IM [CD3^+^CD4^+^CD8α^+^CD8β^−^CD27^+^] in BAL cells. Data represent the mean value of 7–8 pigs ± SEM. Statistical analysis was performed by one-way ANOVA followed by Tukey’s post-test. For head-to-head comparison between two groups, Unpaired t-test was used. Asterisk/Pound represents significant difference between indicated groups (*/♯ *p* < 0.05, **/♯♯ *p*< 0.01, and ***/♯♯♯ *p* < 0.001)

We further characterized the adaptive immune responses in TBLN (Fig. 4A–E) and lungs (Fig. 4F–H). A representative gating strategy to delineate the different activated lymphoid immune cells is shown in Fig. 2B and C. The tumor necrosis factor receptor (TNFR) family member CD27 is a critical co-stimulatory molecule. CD27/CD70 interactions during T-cell-dendritic cell and T-cell-T-cell communications govern T-cell responses both at priming and effector sites esulting in the expansion of effector cells, coupled with improved survivability and differentiation of memory T-cells [24]. Based on the expression of CD27, T-helper/memory cells can be categorized into central memory (CD27^+^) and effector memory (CD27^−^) cells [24]. Central memory T-helper/memory cells display high proliferation ability and intermediate cytokine secretion property. However, their effector memory counterparts exhibit lowest proliferation capacity and highest tendency to generate IFNγ and TNFα [24]. Furthermore, by virtue of age-related changes, and CD27 expression, CTLs can be classified as early effector (CD27^+^) and late effector (CD27^−^) CTLs [7]. We determined the functional ability of antigen-specific T-helper/memory cells and CTLs to generate IFNγ and IL-17A.

Administration of NanoS100-SwIAV-IM vaccine resulted in significantly lower SwIAV H1N1-OH7-specific frequencies of total IL-17A^+^ and IFNγ^+^ CTLs compared to Nano11-SwIAV and NanoS100 groups (*p* < 0.01) (Fig. 4A and C). Furthermore, in case of NanoS100-SwIAV-IM vaccinates, there was a significant downregulation of challenge virus-specific IL-17A^+^ and IFNγ^+^ late effector CTL cell frequencies compared to their non-adjuvanted counterparts and the NanoS100 group animals (*p* < 0.05 to *p* < 0.01) (Fig. 4B and E). Similarly, the frequencies of IFNγ^+^ early effector CTLs were significantly decreased compared to Nano11-SwIAV-IM group animals (*p* < 0.05) (Fig. 4D). However, in case of BAL cells, there was a significant enhancement of the frequencies of challenge virus-specific total CTLs in both NanoS100-SwIAV-ID and Nano11-SwIAV-ID vaccinates compared to NanoS100 [*p* < 0.001] (Fig. 4F). Likewise, both Nano11-SwIAV-ID and NanoS100-SwIAV-ID vaccinates displayed a significant increase in the frequencies of SwIAV H1N1-OH7-specific early effector CTLs compared to NanoS100 group animals (*p* < 0.05 to *p* < 0.001) (Fig. 4G). In contrast, NanoS100-SwIAV IM vaccinates significantly downregulated the frequencies of central memory T-helper/memory cells compared to NanoS100 group animals(*p* < 0.05) in BAL cells (Fig. 4H).

In summary, these data suggested that NanoS100-SwIAV vaccinates exhibited the mobilization and modulation of challenge virus-specific cytokines secreting CTL responses in the lung draining TBLN and lungs of vaccinated pigs at DPC6.

### NanoS100-SwIAV elicited significantly enhanced frequencies of SwIAV-H1N1-OH7-specific IFNγ^+^ effector memory T-helper/memory T-cells, total and late effector CTLs in blood of vaccinated pigs

**Fig. 5:**
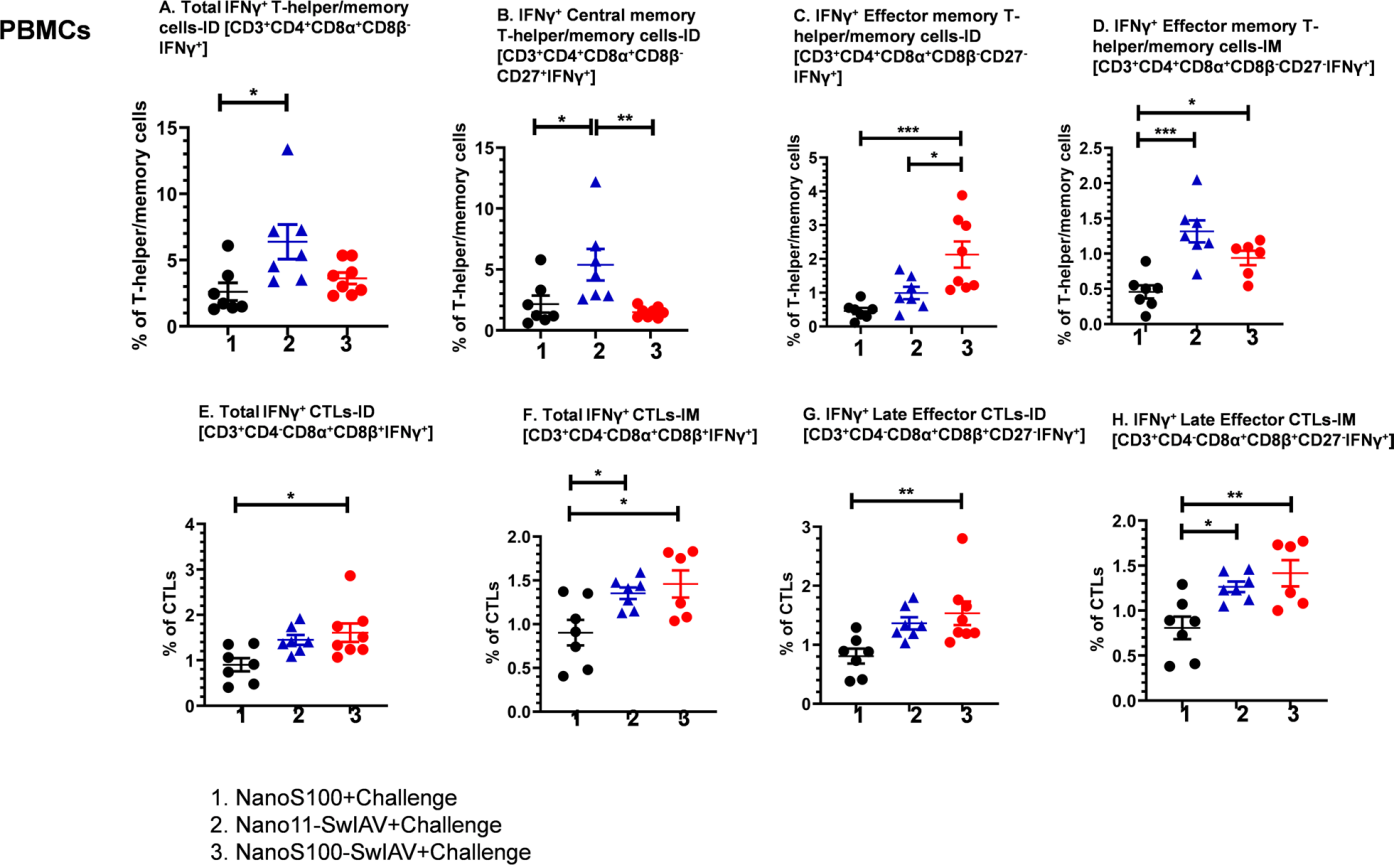
NanoS100-SwIAV induced virus-specific IFNγ^+^ T-helper/memory cells and CTLs in PBMCs of vaccinated pigs. Five-week-old SPF pigs were administered twice ID/IM with NanoS100-SwIAV, Nano11-SwIAV or control NanoS100 and challenged at post-prime vaccination day 35 with SwIAV H1N1-OH7. PBMCs isolated at DPC6 were restimulated with SwIAV H1N1-OH7 in vitro. The cells were labeled and analyzed by flow cytometry for the frequencies of IFNγ^+^ CTLs and T-helper/memory cells: **A** IFNγ^+^ Total T-helper/memory cells-ID [CD3^+^CD4^+^CD8α^+^CD8β^−^IFNγ^+^]; **B** IFNγ^+^ Central memory T-helper/memory cells-ID [CD3^+^CD4^+^CD8α^+^CD8β^−^CD27^+^IFNγ^+^]; **C** IFNγ^+^ Effector memory T-helper/memory cells -ID [CD3^+^CD4^+^CD8α^+^CD8β^−^CD27^−^IFNγ^+^]; **D** IFNγ^+^ Effector memory T-helper/memory cells-IM [CD3^+^CD4^+^CD8α^+^CD8β^−^CD27^−^IFNγ^+^]; **E** Total IFNγ^+^ CTLs-ID [CD3^+^CD4^−^CD8α^+^CD8β^+^IFNγ^+^]; **F** Total IFNγ^+^ CTLs-IM [CD3^+^CD4^−^CD8α^+^CD8β^+^IFNγ^+^]; **G** IFNγ^+^Late Effector CTLs-ID [CD3^+^CD4^−^CD8α^+^CD8β^+^CD27^−^IFNγ^+^]; and **H** IFNγ^+^ Late effector CTLs-IM [CD3^+^CD4^−^CD8α^+^CD8β^+^CD27^−^IFNγ^+^]. Data represent the mean value of 7–8 pigs ± SEM. Statistical analysis was performed by one-way ANOVA followed by Tukey’s post-test. For head-to-head comparison between two groups, Unpaired t-test was used. Asterisk/Pound represents significant difference between indicated groups (*/♯ *p* < 0.05, **/♯♯ *p*< 0.01, and ***/♯♯♯ *p* < 0.001)

We characterized the challenge virus-specific recall cell-mediated immune responses in PBMCs at DPC6. There was a significant enhancement of the frequencies of IFNγ^+^ total T-helper/memory cells and central memory T-helper/memory cells in Nano11-SwIAV-ID vaccinates compared to their counterparts from NanoS100 (*p* < 0.05) (Fig. 5A) and NanoS100-SwIAV-ID groups (*p* < 0.01) (Fig. 5B). However, NanoS100-SwIAV-ID vaccinates exhibited a significant upregulation of IFNγ^+^ effector memory T-helper/memory cell frequencies compared to their counterparts from Nano11-SwIAV-ID and NanoS100 groups (*p* < 0.05 to *p* < 0.001) (Fig. 5C). In contrast, both NanoS100-SwIAV-IM and Nano11-SwIAV-IM vaccinates displayed significantly augmented frequencies of IFNγ^+^ effector memory T-helper/memory cells compared to NanoS100 group animals (*p* < 0.05 to *p* < 0.001) (Fig. 5D). In case of CTLs, there was a significant enhancement of the frequencies of IFNγ^+^ total CTLs in NanoS100-SwIAV-ID vaccinates compared to NanoS100 group (*p* < 0.05) (Fig. 5E). While both NanoS100-SwIAV-IM and Nano11-SwIAV-IM vaccinates exhibited significantly upregulated frequencies of challenge virus-specific IFNγ^+^ total CTLs compared to NanoS100 group (*p* < 0.05) (Fig. 5F). Likewise, there was a significant enhancement of IFNγ^+^ late effector CTLs in case of NanoS100-SwIAV-ID vaccinates compared to NanoS100 group animals (*p* < 0.01) (Fig. 5G). However, both Nano11-SwIAV-IM and NanoS100-SwIAV-IM vaccinates significantly upregulated the frequencies of IFNγ^+^ late effector CTLs compared to NanoS100 group counterparts (*p* < 0.05 to *p* < 0.01) (Fig. 5H). Taken together, NanoS100-SwIAV vaccine elicited strong challenge virus-specific recall IFNγ^+^ T-helper/memory and CTL responses in the peripheral compartment at DPC6.

### NanoS100-SwIAV significantly elicited the SwIAV-H1N1-OH7-specific systemic Th17 response in vaccinated pig

**Fig. 6:**
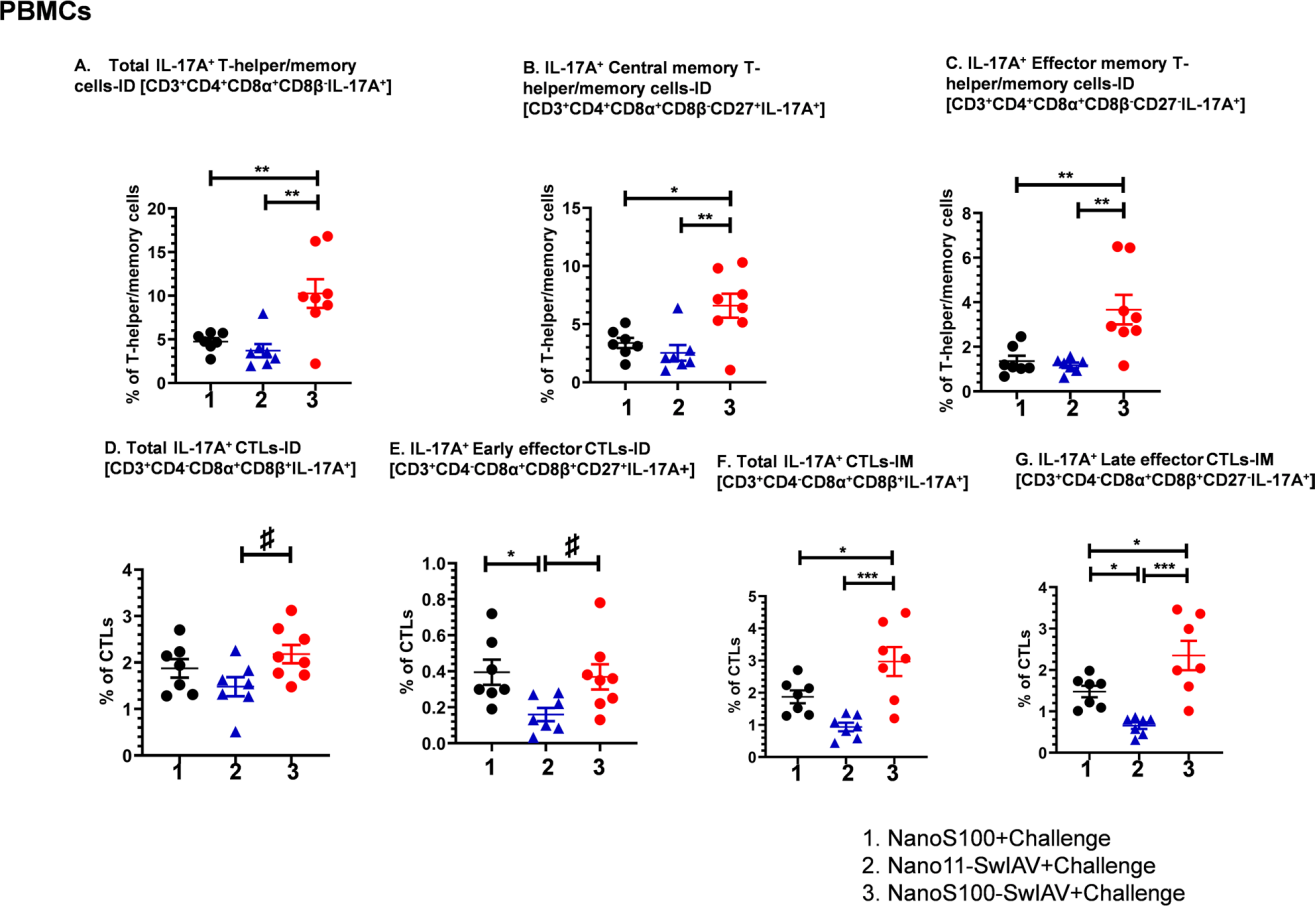
NanoS100-SwIAV elicited virus-specific IL-17A^+^ T-helper/memory cells and CTLs in PBMCs of vaccinated pigs. Five-week-old SPF pigs were administered twice ID/IM with NanoS100-SwIAV, Nano11-SwIAV or control NanoS100 and challenged at post-prime vaccination day 35 with SwIAV H1N1-OH7. PBMCs were isolated at DPC6 and were restimulated with SwIAV H1N1-OH7 in vitro. The cells were labeled and analyzed by flow cytometry for the frequencies of IL-17A expressing CTLs, T-helper/memory cells: **A** Total IL-17A^+^ T-helper/memory cells-ID [CD3^+^CD4^+^CD8α^+^CD8β^−^IL-17A^+^]; **B** IL-17A^+^ Central memory T-helper/memory cells-ID [CD3^+^CD4^+^CD8α^+^CD8β^−^CD27^+^IL-17A^+^]; **C** IL-17A^+^ Effector memory T-helper/memory cells-ID [CD3^+^CD4^+^CD8α^+^CD8β^−^CD27^−^IL-17A^+^]; **D** Total IL-17A^+^ CTLs-ID [CD3^+^CD4^−^CD8α^+^CD8β^+^IL-17A^+^]; **E** IL-17A^+^ Early Effector CTLs-ID [CD3^+^CD4^−^CD8α^+^CD8β^+^CD27^+^IL-17A^+^]; **F** Total IL-17A^+^ CTLs-IM [CD3^+^CD4^−^CD8α^+^CD8β^+^IL-17A^+^]; and **G** IL-17A^+^ Late effector CTLs-IM [CD3^+^CD4^−^CD8α^+^CD8β^+^CD27^−^IL-17A^+^]. Data represent the mean value of 7–8 pigs ± SEM. Statistical analysis was performed by one-way ANOVA followed by Tukey’s post-test. For head-to-head comparison between two groups, Unpaired t-test was used. Asterisk/Pound represents significant difference between indicated groups (*/♯ *p* < 0.05, **/♯♯ *p*< 0.01, and ***/♯♯♯ *p* < 0.001)

We further investigated the challenge virus-specific IL-17A^+^ T-helper/memory and CTL responses in PBMCs at DPC6 (Fig. 6A–G). The NanoS100-SwIAV-ID vaccinates displayed significantly upregulated frequencies of IL-17A^+^ total T-helper/memory cells (Fig. 6A), central memory T-helper/memory (Fig. 6B) and effector memory T-helper/memory cells (Fig. 6C) compared to their non-adjuvanted counterparts (*p* < 0.01) and NanoS100 group animals ((*p* < 0.01).

There was a significant increase of IL-17A^+^ total CTL frequencies in both NanoS100-SwIAV IM and ID vaccinates compared to their non-adjuvanted counterparts (*p* < 0.05 to *p* < 0.001) (Fig. 6D and F). Whereas NanoS100-SwIAV-ID vaccinates exhibited significant augmentation of IL-17A^+^ early effector CTL frequencies compared to Nano11-SwIAV-ID group animals (*p* < 0.05) (Fig. 6E), there was a significant upregulation of IL-17A^+^ late effector CTLs in case of NanoS100-SwIAV IM vaccinates compared to Nano11-SwIAV-IM group (*p* < 0.001) and NanoS100 group (*p* < 0.05) (Fig. 6G). To summarize, the NanoS100-SwIAV vaccine elicited robust SwIAV H1N1-OH7-specific systemic adaptive IL-17A^+^ responses in ID vaccinated pigs at DPC6.

### NanoS100-SwIAV vaccine elicited strong virus-specific IgG responses in the lungs of vaccinated pigs

**Fig. 7:**
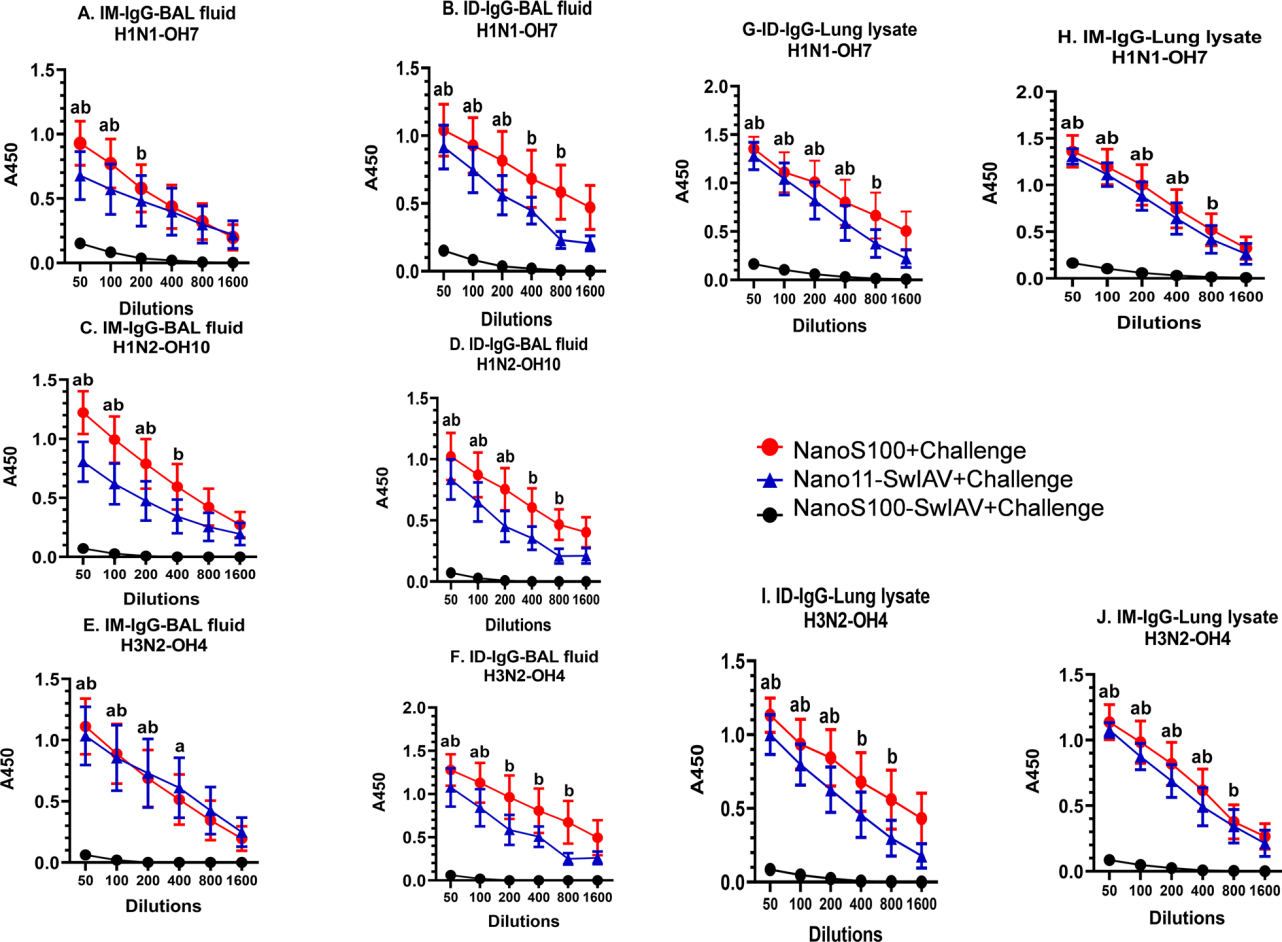
NanoS100-SwIAV enhanced the antigen-specific IgG responses in the lungs of vaccinated pigs. Five-week-old SPF pigs were administered twice ID/IM with NanoS100-SwIAV, Nano11-SwIAV or control NanoS100 and challenged at post-prime vaccination day 35 with SwIAV H1N1-OH7. BAL fluid samples collected at DPC6 were analyzed by ELISA for detecting specific IgG antibody responses against: **A**, **B** H1N1-OH7; **C**, **D** H1N2-OH10; **E**, **F** H3N2-OH4; and in lung lysate samples against **G**, **H** H1N1-OH7; **I**, **J** H3N2-OH4 SwIAV. Data represent the mean value of 7–8 pigs ± SEM. Statistical analysis was performed by two-way ANOVA followed by Bonferroni post-test. Letters a, b and c refer to significance between Nano11-SwIAV + Challenge vs NanoS100 + Challenge, NanoS100-SwIAV + Challenge vs NanoS100 + Challenge, and Nano11-SwIAV + Challenge vs NanoS100-SwIAV + Challenge, respectively, at the indicated sample dilution

Both NanoS100-SwIAV and Nano11-SwIAV vaccinates had significantly increased SwIAV H1N1-OH7-, H1N2-OH10-, and H3N2-OH4-specific IgG antibody levels in BAL fluid compared with nonvaccinated animals regardless of the route of immunization (Fig. 7A–F). Furthermore, analysis of the lung lysate samples revealed a similar trend in case of SwIAV H1N1-OH7 (Fig. 7G and H), and SwIAV H3N2-OH4 (Fig. 7I and J) specific IgG antibodies. These results suggested that both Nano11-SwIAV and NanoS100-SwIAV stimulated significant SwIAV-specific homologous, heterologous and heterosubtypic IgG titers in the lungs of vaccinated animals at DPC6. NanoS100-SwIAV tended to induce higher levels of IgG antibodies compared with the Nano11-SwIAV group, but these differences did not reach statistical significance (Fig. 7A–J).

### NanoS100-SwIAV vaccine induced cross protective systemic virus-specific IgG responses in vaccinated pigs

**Fig. 8:**
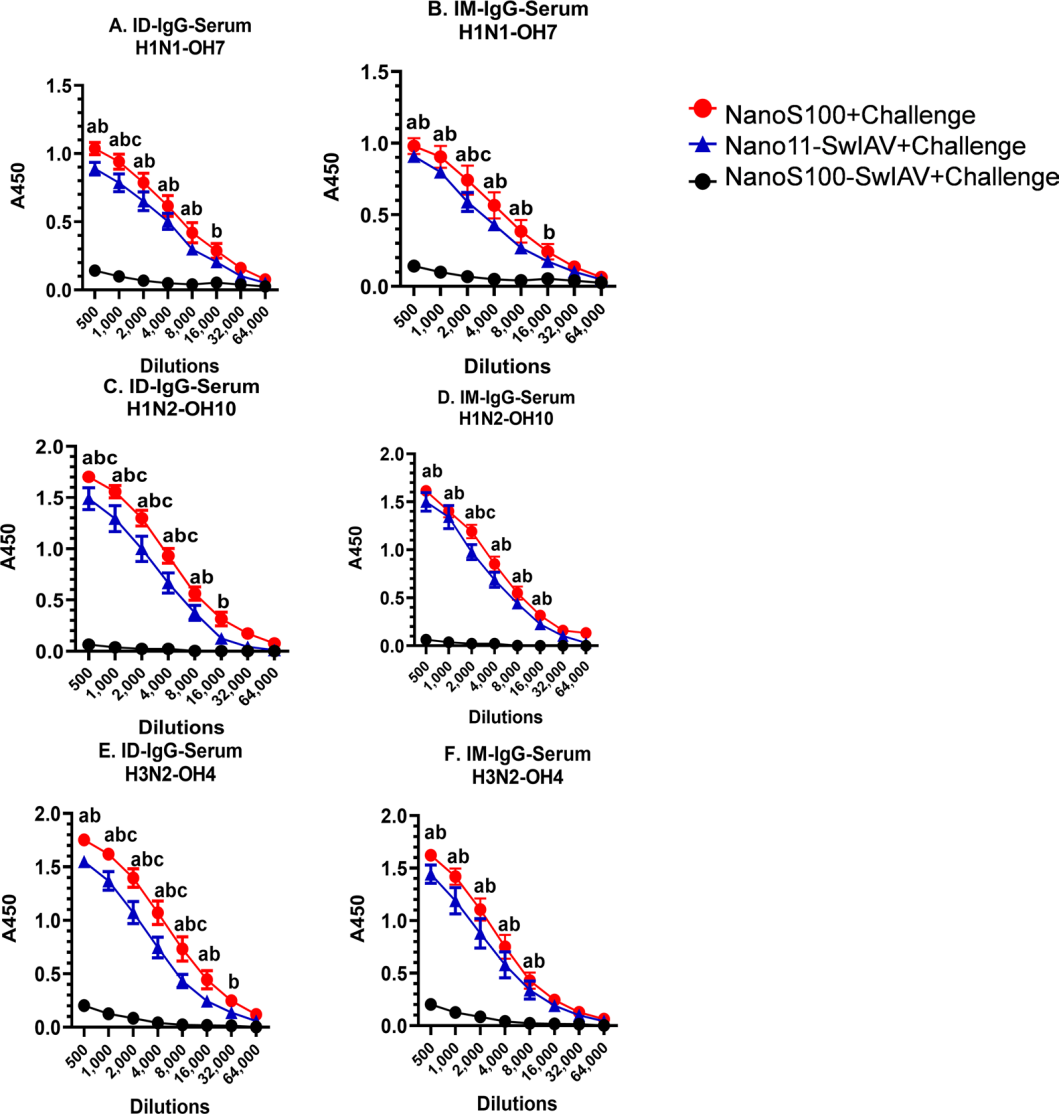
NanoS100-SwIAV elicited antigen-specific IgG responses in the serum of vaccinated pigs. Five-week-old SPF pigs were administered twice ID/IM with NanoS100-SwIAV, Nano11-SwIAV or control NanoS100 and challenged at post-prime vaccination day 35 with SwIAV H1N1-OH7. Serum samples collected at DPC6 were analyzed by ELISA for detecting specific IgG antibody response against: **A**, **B** H1N1-OH7; **C**, **D** H1N2-OH10; and **E**, **F** H3N2-OH4 SwIAV. Data represent the mean value of 7–8 pigs ± SEM. Statistical analysis was performed by two-way ANOVA followed by Bonferroni post-test. Letters a, b and c refer to significance between NanoS100 + Challenge vs Nano11-SwIAV + Challenge, NanoS100 + Challenge vs NanoS100 + Challenge, and Nano11-SwIAV + Challenge vs NanoS100-SwIAV + Challenge respectively, at the indicated dilution

We further characterized the antigen-specific IgG titers in serum of vaccinates. Both Nano11-SwIAV and NanoS100-SwIAV vaccines stimulated significantly higher virus-specific IgG responses against SwIAV H1N1-OH7, H1N2-OH10 and H3N2-OH4 systemically compared to mock vaccinated virus challenged animals (Fig. 8A–F). Furthermore, in NanoS100-SwIAV-ID vaccinates, there was a significant augmentation of SwIAV-H3N2-OH4-specific cross reactive IgG titers in serum compared to their non-adjuvanted vaccinate counterparts (Fig. 8E). Indicating that addition of ADU-S100 led to significant induction of SwIAV-H1N1-OH7, H1N2-OH10 and H3N2-OH4 specific systemic IgG titers in ID vaccinates at DPC6 (Fig. 8A, C and D). Overall, these data suggested that both Nano11-SwIAV and NanoS100-SwIAV vaccines elicited significant homologous, heterologous and heterosubtypic SwIAV-specific IgG responses in the periphery, while the NanoS100-SwIAV-ID elicited significant levels of cross protective serum heterosubtypic SwIAV-specific IgG titers in SPF pigs at DPC6.

### NanoS100-SwIAV vaccine induced significant localized cross protective virus-specific secretory IgA (SIgA) responses in the nasal passage, and lungs of vaccinated pigs

**Fig. 9:**
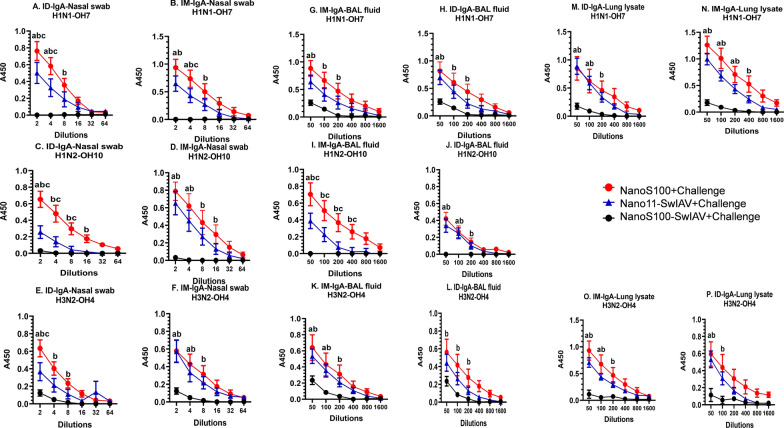
NanoS100-SwIAV elicited antigen-specific localized SIgA responses in respiratory tract and lungs of vaccinated pigs. Five-week-old SPF pigs were administered twice ID/IM with NanoS100-SwIAV, Nano11-SwIAV or control NanoS100 and challenged at post-prime vaccination day 35 with SwIAV H1N1-OH7. Nasal swab samples collected at DPC6 were analyzed by ELISA for detecting specific SIgA antibody responses against: **A**, **B** H1N1-OH7; **C**, **D** H1N2-OH10; **E**, **F** H3N2-OH4; in BAL fluid against **G**, **H** H1N1-OH7; **I**, **J** H1N2-OH10; **K**, **L** H3N2-OH4; and in lung lysate against **M**, **N** H1N1-OH7; and **O**, **P** H3N2-OH4 SwIAV. Data represent the mean value of 7–8 pigs ± SEM. Statistical analysis was performed by two-way ANOVA followed by Bonferroni post-test. Letters a, b and c refer to significance between NanoS100 + Challenge vs Nano11-SwIAV + Challenge, NanoS100 + Challenge vs NanoS100-SwIAV + Challenge, and Nano11-SwIAV + Challenge vs NanoS100-SwIAV + Challenge, respectively, at the indicated dilution

We determined antigen specific SIgA responses in the vaccinated pigs at DPC6. Consistent with the augmented systemic IgG responses in Nano11-SwIAV and NanoS100-SwIAV vaccinates, significantly higher SwIAV H1N1-OH7-, H1N2-OH10-, and H3N2-OH4-specific SIgA titers were observed in nasal swabs compared to their counterparts from NanoS100 group (Fig. 9A–F). Similar trend was evident in BAL fluid samples (Fig. 9G–K) and lung lysates (Fig. 9M–P). Furthermore, NanoS100-SwIAV-ID vaccinates exhibited significantly higher cross protective antigen-specific SIgA titers in nasal passage compared to Nano11-SwIAV-ID (Fig. 9A, C and E). Surprisingly, NanoS100-SwIAV-IM and ID vaccinates displayed significantly higher SwIAV H1N2-OH10-specific SIgA titers in the BAL fluid and nasal swab samples, respectively (Fig. 9I and C). Hence overall, NanoS100-SwIAV was able to stimulate strong localized homologous, heterologous and heterosubtypic SwIAV-specific SIgA titers in the nasal passage and lungs of vaccinated pigs at DPC6.

### NanoS100-SwIAV was effective in eliciting virus neutralizing antibody response in both local and peripheral compartments of vaccinated pigs

**Fig. 10:**
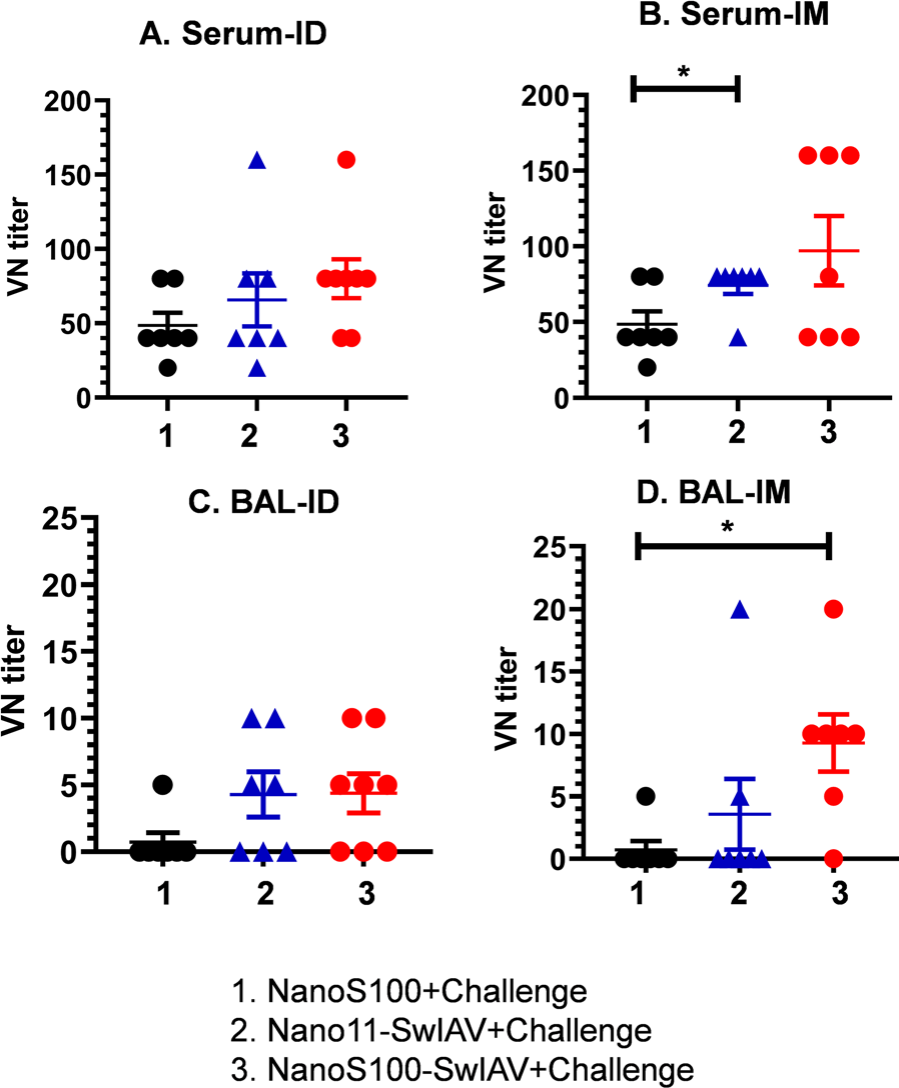
NanoS100-SwIAV induced virus neutralizing antibody responses in both local and peripheral compartments of vaccinated pigs. Five-week-old SPF pigs were administered twice ID/IM with NanoS100-SwIAV, Nano-SwIAV or control NanoS100 and challenged at post-prime vaccination day 35 with SwIAV H1N1-OH7. BAL fluid and serum samples collected at DPC6 were analyzed for estimating challenge virus specific neutralizing antibody titers in: **A**, **B** serum and **C**, **D** BAL fluid by cell culture technique. Data represent the mean value of 7–8 pigs ± SEM

We further probed the antibody responses at the functional level by determining the virus neutralizing antibody titers in the serum and BAL fluid of vaccinated pigs at DPC6. Irrespective of the route of administration, both Nano11-SwIAV and NanoS100-SwIAV vaccines stimulated the challenge SwIAV H1N1-OH7 virus neutralizing antibody secretion in both the BAL fluid and serum of pigs (Fig. 10A–D). Furthermore, NanoS100-SwIAV-IM vaccine elicited significant upregulation of the virus neutralization antibody titers in lungs compared to NanoS100 mock group (*p* < 0.05) (Fig. 10D). Similar trend was observed in case of Nano11-SwIAV-IM vaccinates in blood compared to mock group animals (*p* < 0.05) (Fig. 10B). These results suggested that NanoS100-SwIAV stimulated virus-specific neutralizing antibody responses in vaccinated pigs at DPC6.

### NanoS100-SwIAV induced significantly increased IL-4 and IL-6 cytokine gene expression in TBLN of vaccinated pigs

We characterized the gene expression of different cytokines in TBLN of vaccinates at DPC6. In TBLN MNCs, there was a significant enhancement of IL-6 and IL-4 gene expression in Nano11-SwIAV-IM and NanoS100-SwIAV-IM vaccinates compared to NanoS100 group animals (*p* < 0.05 to *p* < 0.01) (Additional file 1: Fig. S1A and B). Taken together, these data suggest that both Nano-11 and NanoS100 are efficacious in the induction of cytokine responses in the local draining TBLN of vaccinated pigs at DPC6.

## Discussion

Vaccines save lives, and they are one of the greatest public health achievements of the twentieth century [25, 26]. The route of administration, the quality of the adjuvant employed, and nature of the vaccine delivery platform are the critical parameters that govern the efficacy of a vaccine. Our previous studies established the utility and efficacy of plant derived Nano-11, a nanoparticle adjuvant-based vaccine delivering inactivated whole SwIAV by intranasal route in pigs [12, 16]. In the present study, we investigated the performance of split SwIAV H1N2-OH10 antigens adsorbed Nano-11 (Nano11-SwIAV) and adsorbed with ADU-S100 adjuvant (NanoS100-SwIAV) delivered by IM or ID route in pigs.

Intramuscular injection of NanoS100-SwIAV induced complete clearance of challenge virus load from the entire respiratory tract by DPC6. While the intradermal injection of Nano11-SwIAV and NanoS100-SwIAV using a needle-free device significantly cleared (but not complete) the virus from the nasal passage; note that ID vaccinates received half the vaccine dose compared to their IM cohorts. This suggests a potential dose-sparing effect similar to what we previously reported in mice [20]. This is also consistent with a reported randomized open-label trial in young adults, wherein ID vaccination at one-fifth of the standard IM dose of an influenza vaccine induced an immunogenicity profile that was either similar to or better than the IM vaccination [27]. To further delineate the mechanisms underlying the vaccine-induced protection, we dissected the innate and adaptive immune responses in detail.

Chemokine CXCL10 upon induction by interferon-γ functions as a recruiter of target cells expressing CXCR3 transmembrane G protein chemokine receptor. CXCR3 is upregulated in case of Th1 cells. Furthermore, CXCL10 also modulates T-cell development and function [23], and it is a known T cell and monocyte chemoattractant [28]. It is highly induced by stimulation from proinflammatory cytokines (IFNγ and TNFα) [29]. Hence, we investigated the expression of CXCL10 in case of the innate myeloid cells. In the innate immune compartment, NanoS100-SwIAV-ID vaccinates exhibited augmentation of activated myeloid cells in the draining TBLN compared to their non-adjuvanted counterpart ID vaccinates and IM vaccinates (Table 2). While both adjuvanted and non-adjuvanted Nano-11-based IM vaccines elicited significantly higher activated plasmacytoid dendritic cells in lungs and activated myeloid cells in the periphery (Fig. 3). These findings underline the need of investigations to elucidate the role/s of the route of vaccine administration on innate immune mechanisms governing the critical early events leading to the induction of adaptive immune responses induced by ADU-S100 and Nano-11 adjuvants in pigs.

**Table 2:** Summary of significantly upregulated immune cells phenotypes

Immune cell phenotypes	ID Vaccination	IM Vaccination
Activated myeloid cells in TBLN MNCs [CD3^−^CD172^+^CXCL10^+^]	**p < 0.01	
IFNγ^+^ Effector memory T-helper/Memory cells in PBMCs [CD3^+^CD4^+^CD8α^+^CD8β^−^CD27^−^IFNγ^+^]	*p < 0.05	
Total IL-17A^+^ T-helper/Memory cells in PBMCs [CD3^+^CD4^+^CD8α^+^CD8β^−^IL-17A^+^]	**p < 0.01	
IL-17A^+^ Central memory T-helper/Memory Cells in PBMCs [CD3^+^CD4^+^CD8α^+^CD8β^−^CD27^+^IL-17A^+^]	**p < 0.01	
IL-17A^+^ Effector memory T-helper/Memory cells in PBMCs [CD3^+^CD4^+^CD8α^+^CD8β^−^CD27^−^IL-17A^+^]	**p < 0.01	
Total IL-17A^+^ CTLs in PBMCs [CD3^+^CD4^−^CD8α^+^CD8β^+^IL-17A^+^]	^♯^*p* < 0.05	
IL-17A^+^ Early effector CTLs in PBMCs [CD3^+^CD4^−^CD8α^+^CD8β^+^CD27^+^IL-17A^+^]	^♯^*p* < 0.05	
Total IL-17A^+^ CTLs in TBLN MNCs [CD3^+^CD4^−^CD8α^+^CD8β^+^IL-17A^+^]		^##^***p < 0.01
IL-17A^+^ Late effector CTLs in PBMCs [CD3^+^CD4^−^CD8α^+^CD8β^+^CD27^−^IL-17A^+^]		***p < 0.001

Only significantly enhanced immune cell subsets in NanoS100-SwIAV vaccinates compared to their non-adjuvanted counterparts in either ID or IM vaccinated SPF pigs at DPC6 were presented

Asterisk represents significant difference between indicated groups (*/♯ *p* < 0.05, **/♯♯ *p*< 0.01, and ***/♯♯♯ *p* < 0.001)

Our detailed analysis of the challenge virus-specific adaptive cell-mediated memory immune responses in the PBMCs revealed that in NanoS100-SwIAV-ID vaccinates, IFNγ^+^ effector memory T-helper/memory cells, IL-17A^+^ total T-helper/memory cells, IL-17A^+^ central memory and effector memory T-helper/memory cells, total IL-17A^+^ CTLs, and IL-17A^+^ early effector CTLs were significantly enhanced in their frequencies compared to their non-adjuvanted ID vaccinated counterparts; suggesting a robust induction of Th17 and IL-17A^+^ CTL responses in NanoS100-SwIAV-ID vaccinates in the periphery at DPC6. While the frequencies of IL-17A^+^ total CTLs, and late effector CTLs in PBMCs were increased compared to their non-adjuvanted IM vaccinated counterparts (Table 2). These data suggested that ADU-S100 and Nano-11 SwIAV vaccines could upregulate efficient antigen cross-presentation [30] leading to the elicitation of robust antigen-specific CTL responses in pigs. Our data are corroborating the published data reported by other researchers in terms of induction of cell mediated immune responses to influenza virus infection. The SwIAV-specific activated and polyfunctional T cells kinetics were demonstrated in pigs at various days post infection (dpi) [7]. At 4 dpi cycling CD4^+^ T-cells were observed in the TBLN MNCs, at 9 and 12 dpi in PBMCs and TBLN MNCs the enhanced frequencies of polyfunctional IFNγ^+^IL-2^+^TNFα^+^CD4^+^ T-cells were documented. At 44 dpi IFNγ^+^&TNFα^+^, IFNγ^+^&IL-2^+^, and TNFα^+^&IL-2^+^ CD4^+^ T-cells were detected in the lungs, TBLN and blood of SwIAV infected pigs. The major frequencies of SwIAV-specific CD4^+^ T-cells, CD4^+^CD8^+^ T-cells were documented in BAL cells [7]. Furthermore, at dpi 6, we demonstrated the migration of CD8^+^ T-cells to lungs in SwIAV H1N1 infected pigs [31].

Furthermore, both Nano11-SwIAV and NanoS100-SwIAV IM vaccinates exhibited significant expression of IL-4 and IL-6 cytokines in the draining TBLN, and only NanoS100-SwIAV-IM vaccinates exhibited significantly enhanced lymphocyte proliferative response in TBLN MNCs at DPC6. These data indicated vast differences in the expression of different phenotypes of challenge virus-specific CTLs and T-helper/memory cells by Nano11-SwIAV and NanoS100-SwIAV vaccines in the draining TBLN and lungs of vaccinates. IL-6 is a pleiotropic cytokine and there are conflicting reports regarding its role in the context of antiviral immune responses and this aspect needs further investigation [32, 33].

NanoS100-SwIAV stimulated antigen-specific homologous, heterologous, and heterosubtypic SIgA and IgG cross-protective antibody responses within lungs, nasal secretions, and systemically at DPC6. Interestingly, ADU-S100 was able to significantly enhance the SIgA titers within lungs of IM vaccinated pigs at DPC6, and understanding the mechanisms involved in this observation needs further study. Furthermore, irrespective of the route of administration, NanoS100-SwIAV elicited a slightly increased virus neutralizing antibody responses in both local and peripheral compartments in vaccinated pigs, suggesting the important role played by induced cell mediated immune responses.

Our results are consistent with other studies on the split influenza virus vaccines in humans. The split influenza virus vaccine activates monocytes and NK cells through FcγRs leading to the generation of IFNγ but not Type I IFNs. Hence immune complex formation and FcγR activation are most critical in persons with pre-existing IgG against influenza proteins. Detergent-induced splitting will more likely expose additional viral protein epitopes such as matrix proteins. Furthermore, RNA in the split influenza virus vaccine was not able to activate Toll-like receptors suggesting a poor induction of innate immunity [34]. Hence, the addition of powerful adjuvants to split influenza virus vaccines is essential to elicit robust antigen-specific cross-protective responses. In our study, both nanoparticle adjuvant Nano-11 [16, 20] and ADU-S100 are potent adjuvants that acted synergistically with SwIAV H1N2-OH10 split virus antigens leading to the elicitation of cross-protective antigen-specific immune responses. The synthetic STING agonist ADU-S100, by virtue of the higher receptor affinity, upon recognition by the cognate endoplasmic reticulum resident STING receptor, elicits robust type I interferon response [35, 36]. Furthermore, in our study, there was a significant elicitation of IFNγ^+^ CTL response by the Nano-11 and ADU-S100 combination adjuvant suggesting that this combination adjuvant augmented the cross-presentation of SwIAV antigens [30].

In a Phase I randomized controlled trial, a research group investigated the safety, immunogenicity, gene expression, and cytokine responses among AS03-adjuvanted and unadjuvanted inactivated split-virus H5N1 influenza IM vaccines. Employing cell-based systems approach, they characterized the immune responses. Augmentation of serum IL-6 and IP-10 levels were observed within 24 h of AS03-adjuvanted vaccination. Induction of interferon signaling antigen processing and presentation related gene expressions were evident in dendritic cells, monocytes, and neutrophils. In neutrophils, there was enhanced expression of MHC class II antigen-presentation related genes. Furthermore, at 3 days post vaccination, cell cycle genes were upregulated in NK cells, and this correlated with enhanced serum IP-10 levels. These changes were associated with seroprotection at day 56. Taken together, an early augmentation of interferon signaling-related genes predicted the seroprotection 56 days after the first vaccination [37]. In another study the researchers employed split inactivated computationally optimized broadly reactive antigen (COBRA) influenza vaccine in combination with AF03 squalene-in-water emulsion adjuvant elicited protective antibodies against H1N1 and H3N2 influenza viruses in ferrets [38].

The beneficial roles of IL-17A have been demonstrated in mice in the context of BCG vaccination [39], pneumococcal infection in humans [40], *Mycoplasma hyopneumoniae* infection in swine [41], and enterotoxigenic *E. coli* infection and oral immunization with F4 fimbriae in swine [42]. In line with these reports, the results of our study underline the positive effects of antigen-specific IL-17A^+^ cell-mediated responses in case of NanoS100-SwIAV vaccinates. Furthermore, the results of our study corroborate the significance of antigen specific CTLs and T-helper/Memory cell responses against SwIAV [7, 24, 43]. In addition, several studies such as PRRSV infection in pigs [44]; characterization of pseudorabies live attenuated vaccination response [45]; and classical swine fever live attenuated vaccine and virulent challenge infection [46] document the critical role of cross-protective cell-mediated immune responses targeting conserved epitopes. Our overall data suggested that both the Nano11-SwIAV and NanoS100-SwIAV vaccines though induced robust and cross-reactive IgG and IgA antibody responses, the virus neutralizing antibody activity was modest, suggesting a more important role of detected cell mediated Th1 and Th17 immune responses in pigs (Table 2). Future studies are being planned to test the vaccine efficacy using mucosal routes and boost the effectiveness by integration of multivalent SwIAV in combination with powerful adjuvant/s leading to better cross-protection to mitigate the threat of evolving SwIAV field strains. Dissection of the relative contribution of the route of vaccination [47], the adjuvant [48], host microbiota [49] and the vaccine antigen should be performed by employing systems vaccinology approaches and such strategies are expected to yield novel insights required for the rational vaccine design to combat the threat of evolving field strains of swine influenza viruses.

## Conclusions

In spite of a vast genetic difference (77% HA gene identity) between the vaccine SwIAV H1N2-OH10 and the challenge SwIAV H1N1-OH7, the parenterally administered NanoS100-SwIAV vaccine stimulated robust antigen-specific cross-protective cell mediated immune responses, suggesting the potential role of this combination adjuvant in inducing cross-protective immunity against different and evolving influenza viruses in pigs. This is likely caused by the induction of robust cell-mediated immune responses by the NanoS100 combination adjuvant. Additional studies are needed to delineate the specific roles of T cell subpopulations in vaccine-induced protection against swine influenza. These studies demonstrate the utility of this nanoparticle adjuvant in combination with other immunostimulators for different routes of vaccine administration in pigs, and support the further development of Nano-11-based adjuvants for the production of safe and effective veterinary vaccines.

## Methods

### Vaccines and challenge viruses

Binary ethylenimine (BEI) inactivated, detergent (1% Triton-X100 + 0.05% Tween-80) split antigens derived from SwIAV H1N2-OH10 (A/Swine/OH/FAH10-1/10) [50] were used to prepare the Nano-11 based candidate vaccine, while SwIAV H1N1-OH7 (A/Swine/OH/24366/2007) [51] was used for challenge infection. For the characterization of cross-protective antibody responses in vitro, a heterosubtypic IAV H3N2-OH4 (A/Turkey/OH/313053/2004) [52] was used. Viruses were routinely cultured in Madin-Darby canine kidney (MDCK) cells. Nano-11 was prepared from the *Sugary-1* variety of sweet corn as previously described [9]. The Nano-11 based vaccine formulation was prepared using killed SwIAV split antigen SwIAV adsorbed with or without the synthetic STING agonist ADU-S100 (ChemieTek, Indianapolis, IN) electrostatically on Nano-11 leading to two vaccine formulations, NanoS100-SwIAV or Nano11-SwIAV, respectively. A NanoS100 formulation without virus antigens was used as a mock negative control.

### Characterization of NanoS100-SwIAV or Nano11-SwIAV

Adsorption efficiency of ADU-S100 to Nano-11 was quantified by ultraperformance liquid chromatography/tandem mass spectrometry as previously described [20]. Briefly, Nano-11 was mixed with ADU-S100 for 1 h at 4 °C. The mix was centrifuged at 20,800*g* for 15 min at 4 °C. The top supernatant was transferred to a 300 kDa membrane tube (Pall, New York, NY) and centrifuged at 600*g* for 30 min at 4 °C. The filtrate was used to determine the adsorbed concentration of ADU-S100 (MIW815) to Nano-11 with an Agilent 1200 HPLC system connected to an Agilent 6460 Triple-quadrupole mass spectrometer (Agilent Technologies, San Jose, CA). The filtrate solution was recovered and diluted 100 times with a 1:1 (v/v) mixture of acetonitrile and water. A multisampler (G1367D Agilent Technologies, San Jose, CA) was used to deliver 8 µl of the diluted sample to a Waters X-Bridge BEH C18 (3.5 µm particle size, 2.1 × 100 mm column, Waters Corporation, Milford, MA). A flow rate of 0.3 ml/min was used for the high-speed binary pump. Two components comprised the mobile phase: Both A = water with 0.1% formic acid and B = acetonitrile with 0.1% formic acid. Pre-equilibration of the High-performance liquid chromatography column was carried out for one min with 5% of component (B), followed by an 8-min linear gradient to 95% of component (B), which was then held for 2.5 min before being returned to 5% of component (B) for the final 4 min of the pre-equilibration procedure. The samples were examined in negative ion mode with the following parameters for the jet stream electrospray ionization: the capillary voltage is 3500 V, the voltage for fragmentation is 80 V, the nebulizer pressure is 40 pounds per square inch, and the temperature of the gas is 325 °C, the drying gas flow rate is 8 l per min. Multiple reaction monitoring (m/z 688.8 ≥ 133.9, 343.9, and 421.7) was used to quantify ADU-S100 using the following parameters: 20 V collision energy, 30 min of dwell time, and a cell accelerator voltage of 4 W. Standard curves ranging in concentration from 3.9 to 62.5 µg/ml were used to determine the concentration of ADU-S100. The standard curves were fitted using a linear function with an R2 value of 0.99. MassHunter was used for data processing (B.10.00).

Killed SwIAV split antigen was electrostatically adsorbed to Nano-11. A zetasizer (Nano ZS90, Malvern, UK) was used to measure the particle size, polydispersity index (PDI) and zeta potential of Nano-11 ± ADU-S100 ± SwIAV split antigen by dynamic light scattering. The amount of killed SwIAV split antigen adsorbed to Nano-11 ± ADU-S100 was determined by mixing Nano-11 ± ADU-S100 with killed SwIAV split antigen for 1 h at 4 °C. The mix was centrifuged at 20,800*g* for 10 min at 4 °C. The top supernatant was transferred to a 300 kDa membrane tube (Pall, New York, NY) and centrifuged at 600*g* for 30 min at 4 °C. The filtrate was used to test killed SwIAV split antigen adsorption to Nano-11 ± ADU-S100. Protein adsorption efficiency was determined by subtracting the unbound killed SwIAV split antigen in the supernatant from the initial amount used for adsorption using a micro-BCA protein assay kit (Thermo Fisher Scientific, Waltham, MA).

### Animal studies

Piglets were delivered by Cesarian-section and raised in our BSL-2 facility. Five-week-old SPF pigs were injected twice ID [each 0.1 ml dose consisted of 0.5 mg Nano-11 + 25 µg ADU-S100 + 125 µg viral protein comprising of 25 hemagglutination (HA) units] or IM [each 0.5 ml dose consisted of 1 mg Nano-11 + 50 µg ADU-S100 + 250 µg viral protein comprising of 50 HA units] with NanoS100-SwIAV, Nano11-SwIAV, or control Nano11S100 and challenged at post-prime vaccination day 35 with SwIAV H1N1-OH7 [2 × 10^7^ the median tissue culture infective dose (TCID_50_/ml) per pig; half the dose intratracheal and half the dose intranasal] (Table 3). The ID injections were performed with a needle-free injector (Tropis^®^, Pharmajet, Golden, CO). Nasal swab and blood samples were collected on day post vaccination 0 (DPV0) and DPV21. The pigs were sacrificed on day post challenge (DPC) 6 and nasal swab, blood, lungs, TBLN, and bronchioalveolar lavage (BAL) were collected for the analysis. Nasal swabs samples were also collected on DPC0, DPC2 and DPC4.

**Table 3:** Experimental design to evaluate NanoS100-SwIAV vaccine efficacy in pigs


Pig Groups	Route	Number of pigs
NanoS100 + Challenge	IM	4
NanoS100 + Challenge	ID	3
Nano11-SwIAV + Challenge	ID	7
NanoS100-SwIAV + Challenge	ID	8
Nano11-SwIAV + Challenge	IM	7
NanoS100-SwIAV + Challenge	IM	7

Five-week-old SPF pigs were administered twice ID/IM with NanoS100-SwIAV, Nano11-SwIAV or control NanoS100 and challenged at post-prime vaccination day 35 with SwIAV H1N1-OH7 and euthanized day post challenge [DPC] 6. Nasal swab, blood, lungs, TBLN, BAL cells were collected for the analysis. Three-eight pigs were used in each of the experimental groups

IM, Intramuscular; ID, Intradermal; NanoS100, Nano11 + ADU-S100; SwIAV, Swine influenza A virus H1N2 split antigen; Challenge, SwIAV H1N1 OH7

### Analysis of antigen-specific T-cell responses

Peripheral blood mononuclear cells (PBMCs), TBLN mononuclear cells (MNCs), and BAL cells were restimulated in the presence of recombinant porcine IL-2 and SwIAV H1N1-OH7 at 0.1 multiplicity of infection (MOI) for 48 h in vitro. The cells were labeled and characterized by flow cytometry for the frequencies of different types of myeloid and lymphocyte subsets such as cytotoxic T-lymphocytes (CTLs) [CD3^+^CD4^−^CD8α^+^β^+^], T-helper/memory cells [CD3^+^CD4^+^CD8α^+^β^−^], and naïve T-helper cells [CD3^+^CD4^+^CD8α^−^β^−^CD27^+^].

### Surface and intracellular cytokine staining for flow cytometry

PBMCs, TBLN MNCs, and BAL cells were isolated and labeled as described previously [6, 12, 53]. Briefly, 5 million cells were seeded in a 48-well flat bottom plate in 1 ml total volume of culture medium (RPMI 1640 10% FBS) in the presence of recombinant porcine IL-2 and restimulated with SwIAV H1N1-OH7 for 48 h (hrs) in vitro. Protein transport inhibitor Brefeldin A (GolgiPlug) was added for the last 6 h of the incubation period. Cells were harvested, washed, blocked with 1% normal rabbit serum, and divided into appropriate number of wells in a 96-well round bottom plate for surface and intracellular cytokine labeling. Appropriate isotype control antibody staining was included as negative control for the flow cytometry. The antibody panels and their corresponding isotype controls used in flow cytometry were described (Additional file 2: Tables S1–S3). If in the FACS panel, any purified/unlabeled mAb is present, the cells were first labeled with purified mAb and its corresponding secondary antibody followed by blocking with 1% normal mouse serum. This step was followed by staining for other cell markers together. Cells were transferred to a 96-well round bottom plate, washed twice in 200 µl FACS buffer (Hank’s Balanced Salt solution, 1% BSA 0.02% sodium azide)/well, and subjected to surface labeling using fluorochrome conjugated monoclonal antibodies (mAbs) against indicated markers and their corresponding isotype controls at pre-titrated concentrations in 50 µl of FACS buffer for 30 min at 4 °C. Cells were then fixed using 1% paraformaldehyde at 4 °C for 30 min and resuspended in FACS buffer.

For intracellular staining cells were washed once with FACS buffer and permeabilized with 1% saponin for 45 min at room temperature. Subsequently, cells were washed with saponin wash buffer (0.1% saponin) and incubated with fluorochrome conjugated mAbs against indicated markers and their corresponding isotype controls at previously titrated and optimized concentrations in 50 µl final volume in saponin wash containing 1% normal rabbit serum for 45 min at 4 °C. Cells were washed once in saponin wash and stained with indicated secondary antibodies for 45 min at 4 °C. Cells were washed once and resuspended in 200 µl FACS buffer and transferred to FACS tubes and acquired using a lymphocyte gate by using BD FACS Aria II flow cytomer. For each sample, 100,000 events were acquired. The data were analyzed using FlowJo software (FlowJo V10, Becton, Dickinson& Company; BD) and plotted using GraphPad Prism (GraphPad Prism 9, CA).

### Enzyme-linked immunosorbent assay (ELISA)

SwIAV-specific isotype IgG and secretory IgA (SIgA) antibodies titers were determined by ELISA as described previously [53, 54]. Briefly, 96-well flat bottom high binding affinity plates were coated with pre-titrated (10 μg/ml) killed virus antigens of SwIAV H1N1-OH7, H1N2-OH10, or H3N2-OH4 and incubated overnight at 4 °C. Plates were washed with PBS-Tween20 (0.05%) (PBST) and blocked for 2 h with 5% dry milk in PBST. Test samples were serially diluted in 2.5% dry milk powder in PBST at a starting dilution of 1:2 for nasal swab and 1:100 for serum, BAL, and lung lysate samples, and 50 μl/well were added to plates and incubated overnight at 4 °C. This step was followed by washing and incubation at room temperature (RT) for 2 h with 50 μl/well of goat anti-pig IgA conjugated with HRP (Bethyl Laboratories Inc., TX) at pre-titrated 1:2,000 dilution or peroxidase labeled AffiniPure Goat Anti-Swine IgG (H + L) (Jackson ImmunoResearch Laboratories Inc., PA) 1:8000 dilution in 2.5% dry milk in PBST. After washing the plates, a 1:1 mixture of peroxidase substrate solution B and TMB (KPL, MD) (50 μl/well) was added and incubated for 10–20 min at RT. The reaction was stopped by adding 1 M phosphoric acid (50 μl/well) and the optical density (OD) was measured by Spectramax microplate reader at 450 nm. The corrected OD values were obtained by subtracting the average value of blank from the test samples.

### Virus titration

Virus titers were determined as previously described [53, 54]. Briefly, 96-well tissue culture plates were seeded with MDCK cells (2 × 10^4^ cells/well) in 200 μl of DMEM enriched media and incubated in a 37 °C humidified 5% CO_2_ incubator overnight. In another set of sterile 96-well round-bottom plates, a ten-fold serial dilution of nasal swab, BAL fluid, and lung lysate samples were prepared using serum-free DMEM. Plates containing confluent monolayer of MDCK cells were washed three times with sterile 1X PBS and inoculated with 100 μl/well of serially diluted test samples for 1.5 h at 37 °C in a 5% CO_2_ incubator followed by 100 μl/well of DMEM serum-free medium containing 2 µg/ml TPCK-trypsin (Sigma, St. Louis, USA) were added. This step was followed by incubation in 37 °C humidified 5% CO_2_ incubator for 36 h for the development of cytopathic effect (CPE) and fixed with 80% acetone (10 min) for immunostaining. Cells were incubated with (50 μl/well) an IAV nucleoprotein specific mAb (#M058, CalBioreagents, CA) diluted 1:5000 for 2 h at 37 °C in a 5% CO_2_ incubator. This was followed by washing and incubation for 1.5 h with (50 μl/well) the secondary antibody Alexa Fluor 488 conjugated goat anti-mouse IgG (H + L) antibody (Life Technologies, OR) at 37 °C in a 5% CO_2_ incubator. Mounting media (Glycerol: PBS, pH = 8 at 6:4 proportion) 50 μl/well was added to the immunostained cell plates. Infection was recorded using an Olympus, NY fluorescent microscope, and infectious titer was determined by Reed and Muench method.

### Statistical analysis

Statistical analysis of immune cells data was carried out by using one-way ANOVA followed by Tukey’s post-test. There were no statistical differences between the two control groups (IM and ID adjuvant only), and these were combined into one control group for analysis versus the IM and ID immunized groups. For comparison between two groups, unpaired t-test was used. Analysis of titers of IgG and SIgA antibody responses were carried out using two-way ANOVA followed by the Bonferroni test. Data represent the mean value of 7–8 pigs ± SEM.

## Supplementary Information

**Additional file 1: Figure S1.** Nano11-SwIAV induced IL-4 and IL-6 cytokine gene expression in TBLN of vaccinated pigs. Five-week-old SPF pigs were administered twice ID/IM with NanoS100-SwIAV, Nano11-SwIAV or control NanoS100 and challenged at post-prime vaccination day 35 with SwIAV H1N1-OH7. TBLN samples collected and stored in RNAlater at DPC6 were analyzed for gene expression using SYBR Green qRT-PCR method. Data represent the mean value of 7-8 pigs ± SEM. Statistical analysis was performed by one-way ANOVA followed by Tukey’s post-test. Asterisk represents significant difference between indicated groups (*p < 0.05, **p < 0.01).

**Additional file 2: Tables S1–S3.** Antibody panels used in the flow cytometry analysis. The antibody panels employed for the analysis of T-helper/memory cells, CTLs, and myeloid cells in TBLN MNCs, PBMCs, and BAL cells in Panel#1[Table S1] (Myeloid cells); IL-17A^+^ lymphocyte subsets in Panel#2 [Table S2]; and IFNγ^+^ lymphocyte subsets in Panel#3 [Table S3].

## Data Availability

The datasets used and/or analysed during the current study are available from the corresponding author on reasonable request.
