# Correction: A split influenza vaccine formulated with a combination adjuvant composed of alpha-d-glucan nanoparticles and a STING agonist elicits cross-protective immunity in pigs

**DOI:** 10.1186/s12951-022-01741-x

**Published:** 2022-12-23

**Authors:** V. Patil, J. F. Hernandez-Franco, G. Yadagiri, D. Bugybayeva, S. Dolatyabi, N. Feliciano-Ruiz, J. Schrock, J. Hanson, J. Ngunjiri, H. HogenEsch, G. J. Renukaradhya

**Affiliations:** 1 Center for Food Animal Health, Department of Animal Sciences, The Ohio State University, 1680 Madison Avenue, Wooster, OH 44691 USA; 2 Department of Comparative Pathobiology, College of Veterinary Medicine, Purdue University, West Lafayette, IN USA; 3 International Center for Vaccinology, Kazakh National Agrarian Research University (KazNARU), Almaty, Kazakhstan

**Correction: Journal of Nanobiotechnology (2022) 20:477** **https://doi.org/10.1186/s12951-022-01677-2**

Following publication of the original article [1], the authors would like to make a change in the colour of figure legends for the figures 7, 8 and 9. 
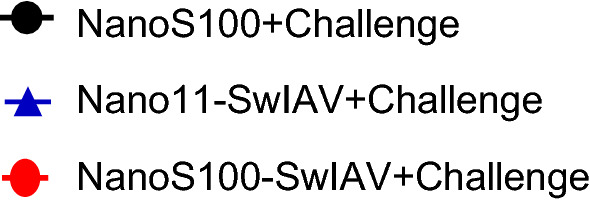


The original article [1] has been corrected.
